# Roof Shape Classification from LiDAR and Satellite Image Data Fusion Using Supervised Learning

**DOI:** 10.3390/s18113960

**Published:** 2018-11-15

**Authors:** Jeremy Castagno, Ella Atkins

**Affiliations:** Robotics Program, University of Michigan, Ann Arbor, MI 48109, USA

**Keywords:** geographical information system (GIS), LiDAR, machine vision, machine learning, unmanned aircraft systems (UAS), drones, maps, safety

## Abstract

Geographic information systems (GIS) provide accurate maps of terrain, roads, waterways, and building footprints and heights. Aircraft, particularly small unmanned aircraft systems (UAS), can exploit this and additional information such as *building roof structure* to improve navigation accuracy and safely perform contingency landings particularly in urban regions. However, building roof structure is not fully provided in maps. This paper proposes a method to automatically label building roof shape from publicly available GIS data. Satellite imagery and airborne LiDAR data are processed and manually labeled to create a diverse annotated roof image dataset for small to large urban cities. Multiple convolutional neural network (CNN) architectures are trained and tested, with the best performing networks providing a condensed feature set for support vector machine and decision tree classifiers. Satellite image and LiDAR data fusion is shown to provide greater classification accuracy than using either data type alone. Model confidence thresholds are adjusted leading to significant increases in models precision. Networks trained from roof data in Witten, Germany and Manhattan (New York City) are evaluated on independent data from these cities and Ann Arbor, Michigan.

## 1. Introduction

Geographic information system (GIS) data are openly available for a variety of applications. Data on terrain height and type have historically been available, with high-accuracy labeled data now increasingly available, e.g., building footprints and heights. Systematic characterization of building roof architecture and slope offers a new dimension to traditional terrain data. These data could be used to rapidly identify building change or damage from the air, to improve in-flight localization capabilities in GPS-denied areas, and to inform small Unmanned Aircraft Systems (UAS) of alternative ditching sites, a problem previously investigated by the authors [1,2]. Databases such as OpenStreetMap (OSM) [3] provide limited roof information, but such data have been manually entered to-date thus is sparse.

**Figure 1:**
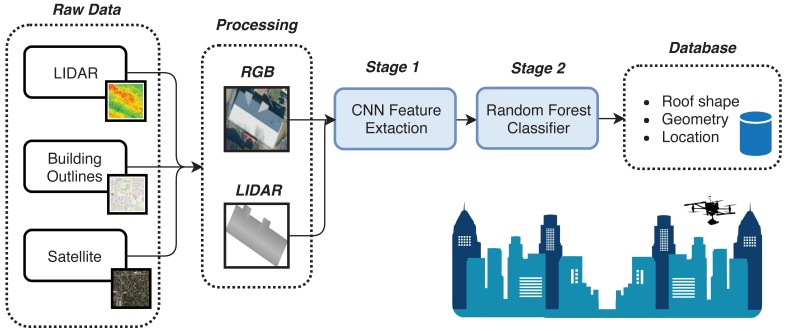
Roof classification data fusion and processing pipeline. LiDAR, building outlines, and satellite images are processed to construct RGB and LiDAR images of a building rooftop. In Stage 1, these images are fed into a CNN for feature extraction, while Stage 2 uses these features with a random forest for roof classification. These data can be stored for quick reference, e.g., navigation or emergency landing site purposes.

This paper fuses satellite imagery and airborne Light Detection and Ranging (LiDAR) data through multiple stages of machine learning classifiers to accurately characterize building rooftops. With these results, roof geometries worldwide can be stored in an easily-accessible format for UAS and other applications. Supervised training datasets are automatically generated by combining building outlines, satellite, and LiDAR data. The resulting annotated dataset provides individual satellite image and LiDAR (depth) image representations for each building roof. Roof shapes are automatically categorized through a novel combination of convolutional neural networks (CNNs) and classical machine learning. Transfer learning is employed in which multiple pre-trained CNN model architectures and hyper-parameters are fine-tuned and tested. The best performing CNN for both satellite and LiDAR data inputs is used to extract a reduced feature set which is then fed into either support vector machine (SVM) or random forest classifiers to provide a single roof geometry decision. Validation and test set accuracies are evaluated over a suite of different classifier options to determine the best model(s). A range of urban environments are used to train and test the proposed models. Data from Witten, Germany; Ann Arbor, Michigan; and the Manhattan borough of New York City, New York are collected and manually labeled to represent small to large metropolitan city centers. We show that combining datasets from both small and large cities leads to a more generalized model and improves performance. Figure 1 provides an overview of the data processing pipeline and illustrates a UAS localization and contingency landing use case [2].

Although this is our first journal publication on roof classification, this paper extends our preliminary work presented at a conference [4] on both processing methods and application to diverse geographical and architectural environments. Specific contributions include:

- Over 4500 building roofs spanning three cities have been manually classified and archived with a satellite and LiDAR depth image pair. This dataset is released with this paper.
- New “complex-flat” and “unknown” roof shape classes enable the machine classifier to distinguish flat roofs with infrastructure (e.g., air conditioning and water towers), unfamiliar roof shapes, and images of poor quality.
- This paper significantly reduces the set of outliers that previously required manual removal for training and test datasets (from 45% in [4] down to 5% in this paper). This paper’s test set accuracies represent a reasonable expectation of results when deployed in new areas.
- An analysis of confidence thresholding is presented to improve the model’s predictive power. This ensures only correct labels are assigned which is critical for use in high risk scenarios.
- Expanded results are presented from use of a single trained classifier (over Witten and Manhattan) tested with datasets from three cities, one of which (Ann Arbor) was never used for training or validation.

The paper is structured as follows. First, GIS data sources and prior roof geometry classification work are summarized. Next, background in machine learning and data extraction methods is provided. Specific methods to extract data for input to this paper’s machine learning feature extraction and classification system are presented, followed by a description of training, validation, and test runs performed. Statistical accuracy results are presented followed by a discussion and conclusions.

## 2. Background

This section summarizes related work. First, GIS data sources and previous efforts to extract roof geometries are reviewed. Next, convolutional neural networks (CNNs) and their application to feature extraction are reviewed.

### 2.1. Roof Geometry Classification

Satellite color images and 3D point cloud data from airborne LiDAR sensors provide complementary roof information sources. High resolution satellite images offer rich information content and are generally available worldwide. However, extracting 3D building information from 2D images is difficult due to occlusion, poor contrast, shadows, and skewed image perspectives [5]. LiDAR point clouds provide depth and intensity measurements that capture the features of roof shapes, yet LiDAR does not offer other world feature information from ambient lighting intensity and color. LiDAR point cloud data are often processed and converted to digital surface models (DSM) representing the top surface layer of any terrain.

The amount of detail desired for roof geometry influences data processing methods. Detailed reconstruction of 3D city maps for visualization or simulation purposes often requires a detailed representation of the geometric elements in a 3D building model. This is often accomplished using a model based or data driven approach. In a model-based approach, a collection of parameterized building models are selected as possible candidates given prior knowledge of buildings in the geographic region of interest. Buildings are then fit to these models using the gathered data points, and the best 3D model is chosen. This method can reliably extract parameters from data points so long as the building shape is simple and roof details are not required [6]. A data-driven approach does not require a priori knowledge of building structures, instead using large datasets to generate a high-fidelity model. Data points are grouped to define planar surfaces which in turn are used to construct 3D lines fully specifying building geometry. For example, work by Jochem et al. [7] segments potential roof points in a building through their normal vectors, which are later collapsed into planar elements that conform to the defined constraints of roof planes.

The photogrammetry community has demonstrated recent success in applying data driven approaches for 3D building reconstruction. Yan et al. [8] proposed a dynamic multi-projection-contour (DMPCA) framework that uses super generalized stereo pairs (SGSP) to generate and iteratively refine 3D buildings models. This method minimizes the total difference between the projection-contour of a building across SGSPs and the projection-contours of the simulated 3D model. Using building images captured by a UAS, Malihi et al. [9] generated a dense point cloud from image matching. This point cloud is then clustered by RANSAC shape detection. Planar geometry is then determined through least squares fitting, and finally refined details (e.g., dormers and eaves) are modeled. Maset et al. [10] proposed the use of both thermal infrared (TIR) and RGB images taken by UAS to generate point clouds. These distinct point clouds are then aligned with an iterative closest point (ICP) procedure generating a high fidelity building model with accompanying RGB textures. Similarly, Yan et al. [11] proposed a roof-contour and texture-image guided interpolation (RTGI) method that generates facades as well as texture maps of buildings. A common theme in most of the above research is the increased use of UAS to capture high resolution data from multiple viewpoints to improve model accuracy.

The localization and landing site applications for UAS referenced by this paper only require a simple classification of building roof shape. In fact, complex model representations are undesirable given that UAS applications would be computed by a low-power lightweight embedded processor. Classical machine learning algorithms such as support vector machines (SVM), logistic regression, and decision trees are often used in these classification scenarios but invariably face computational complexity challenges caused by the high dimensionality found in these GIS data sources. To employ these algorithms, a reduction in dimensionality through feature selection is often performed. Recent work by Mohajeri et al. [12] performed roof classification through SVM’s by reducing a DSM image of a roof to a set of handcrafted features such as the number of roof surfaces for each building and the distribution of the binned slope angles. A set of 717 buildings in Geneva, Switzerland were manually labeled for training and testing purposes of the model, resulting in an overall accuracy of 66% for a six roof type classification. The same authors also experimented using a random forest classifier with similarly handcrafted features from a DSM on a 1252 building dataset from Switzerland. The test set was a 25% random sampling of the labeled dataset with a reported total accuracy of 70% when identifying six roof types [13].

Recent advances with deep learning with techniques such as convolutional neural networks (CNN) have demonstrated the ability to accurately and robustly classify high dimensional data sources such as camera images [14]. The GIS community has begun to apply CNNs to roof identification. Perhaps most closely related to this paper, Alidoost and Arefi [15] trained CNNs using satellite red green blue (RGB) imagery and digital surface map (DSM) images to label basic roof shapes. However, the final predicted roof shape was simply taken as the highest probability result between the two models (RGB, DSM); no feature fusion or training was performed between different modalities. Training and test set sizes are not explicitly provided, however two test set accuracies are reported: 95% and 88% using the the authors’ best model. Complementary work by Partovi et al. [16] fine-tuned a CNN using patched satellite images of building rooftops. Using the fine-tuned CNN, the authors extracted high-level features of images as inputs to a second-stage SVM classifier. Approximately 3000 images in Munich, Germany were used for training and testing resulting in 76% total accuracy. Our paper adopts an analogous two-stage processing approach to roof classification with the novel addition of LiDAR and satellite image feature fusion. Specifically, this fusion allows the creation of a nonlinear decision function that exploits the strengths of each modality. Finally, unlike all previous work we have encountered, this paper incorporates data from geographically diverse cities and assesses models on their ability to generalize across regions.

### 2.2. The Convolutional Neural Network (CNN)

**Figure 2:**
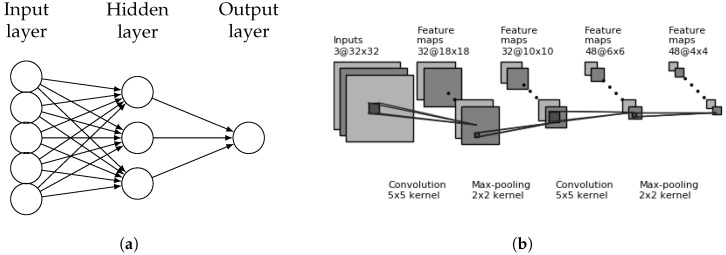
(**a**) Example of a fully connected neural network with one hidden layer. (**b**) Example of a CNN with two convolutional blocks.

An artificial neural network is composed of a series of functional layers connected in a weighted graph structure. Each neural network layer consists of a *node* vector, a node activation function, and weighted edges typically feeding forward to the next network layer. A layer is considered fully connected (FC) if every node in the layer is connected to every node in the previous layer. Repeating layers are called blocks and can have unique structural and functional designs. An example is shown in Figure 2a.

Convolutional neural networks (CNNs) are primarily distinguished by their shared weights and translation-invariance characteristics. CNNs hold multiple convolutional blocks that are generally composed of a convolutional filter layer, an activation layer, and finally a pooling or downsampling layer. These blocks generate high level features from their inputs which are then fed into the next set of blocks. Figure 2b shows an example of an input image passing through two convolution blocks. Eventually, a final feature set is produced which feeds into fully-connected layers generating an output feature vector or classification. The dimensions of CNN blocks and how they interconnect with each other and subsequent layers determines the *architecture* of the network. Researchers have developed several CNN architectures that have been tested against large image sets such as Imagenet [17]. These networks are trained from scratch, meaning their weights are randomly initialized, and take weeks (of real-time) to converge even with the aid of general purpose graphics processing units (GPGPUs). For example, the Large Scale Visual Recognition Challenge 2012 (ILSVRC2012) holds a dataset of over a million images with the task of distinguishing between 1000 categories. CNN classifiers achieved “Top 5” accuracies of greater than 95%.

For a CNN to be applied to an application such as roof classification, a large supervised domain-specific training set is needed. If a large training dataset is not available, a technique called transfer learning can be applied. Transfer learning accelerates machine learning by transferring knowledge from a related, perhaps generalized, domain to a new domain [18]. This technique requires the use of an existing *pre-trained* CNN. The beginning layers of the pre-trained CNN often generate domain-independent features (e.g., features which distinguish lines or color changes) that will be useful for other domains. The base architecture and associated weights are used as the starting layers in a new CNN to be trained. An opportunity also arises during the training process to *freeze* a variable number of initial layers’ weights, thereby reducing the number of parameters to learn and overall training time. In essence, the more initial layers that are frozen, the more the CNN relies upon the pre-trained model’s domain knowledge.

In addition to transfer learning, image augmentation (rotation, cropping, etc.) can be used to artificially inflate the training dataset, which tends to reduce overfitting. Parameters such as the size of the fully connected layers or number of frozen initial layers influence the accuracy of the model. Optimal parameters are determined by evaluating multiple trained networks against a validation set and assessing its accuracy. Parameter adjustments are grouped as hyperparameters to determine an optimal model structure.

### 2.3. Feature Extraction and Classical Machine Learning

**Figure 3:**
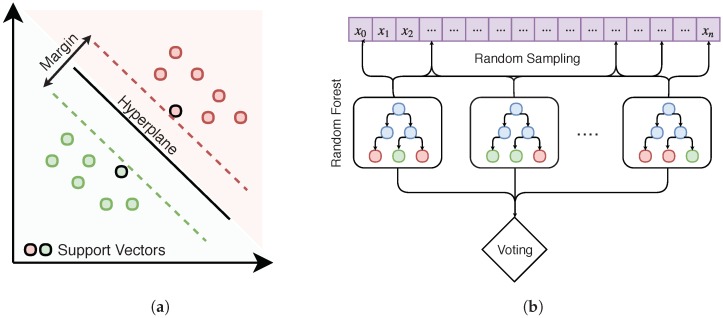
(**a**) Example of an SVM separating two classes with a hyperplane. Optimal class separation is guaranteed by maximizing margin size. (**b**) Example of a random forest with multiple decision trees being trained on random samples from the training data.SVM and Random Forest

Supervised learning classification algorithms such as support vector machines (SVM) and decision trees have difficulty handling large GIS datasets such as images or point clouds. However, when given a reduced feature set, both approaches can be effective for final classification [12,19]. Researchers have begun to use CNN’s to extract a “Stage 1” reduced feature set that is then fed into a downstream “Stage 2” classifier. Support vector machines (SVM) divide a feature space into linear hyperplanes for class separation, but often use kernels to project input features into higher-dimensional spaces to create non-linear decision boundaries. The best kernel to be used is dependent upon the feature set provided; however, linear, polynomial, and radial based function (rbf) kernels are often the first used. Figure 3a shows an SVM separating a binary class (red/green) with the line that maximizes margin distance between classes; a linear kernel is used. Similarly, random forest classifiers create nonlinear decision boundaries through ensemble learning, a technique that trains many decision trees on random subsets of the training data as shown in Figure 3b. The forest is represented by the many decision trees created and trained, and the final classification is the statistical mode of the trees’ collected predictions. The forest is often limited by the number of trees (i.e., number of estimators) as well as the maximum depth of any tree in its collection. Random forest classifiers are resilient to overfitting through the collected knowledge of the ensemble. This paper will train both SVM and random forest classifiers on CNN extracted features from satellite and LiDAR building images in an effort to improve classification accuracy.

## 3. GIS Data Processing, Image Generation, and Training

Section 3.1 details the process of generating an annotated dataset and its random split into distinct training, validation, and testing subsets. Section 3.2 and Section 3.3 outline image generation techniques from LiDAR and satellite data, respectively. Section 3.4 details the specific CNN architectures and training procedures, followed by validation assessment. Section 3.5 explores CNN feature extraction as input for several chosen classical machine learning algorithms and their associated parameters.

### 3.1. Classified Image Set Generation

Generation of an annotated roof dataset requires three data sources for each building: satellite imagery, airborne LiDAR data, and building outlines with corresponding roof labels (from manual classification). Buildings outlines are used to extract individual roofs from satellite and LiDAR data. Using building outlines to filter such data sources is a technique used within the GIS community [20,21]. For example, Tack et al. [22] used 2D cadastral maps to clip buildings from a DSM for 3D building reconstruction. This clipping step allows for the subsequent generation of images focused on the building of interest and enhances feature extraction.

All three of these data sources must be properly geo-referenced so they can be fused together. Care must be taken to select a geographic area where data sources for all of these items are present. Although OSM provides the necessary building outlines in many geographic regions, the associated roof shape label is most often incomplete. Some geographic regions (e.g., Germany) are more likely to have a denser collection of labeled roof shapes through a higher volunteer involvement. Previous work by the authors relied upon pre-labeled roof shapes provided by the OSM database [4] in Witten, Germany. However, this paper broadens the categories of classifiable roof shapes as well as sampling from diverse regions including small to large city centers. The authors found that OSM did not provide sufficient pre-labeled buildings, necessitating manual classification of thousands of roof shapes (by the first author). Once the appropriate data sources are found or generated, the methods described below can be employed to generate satellite and LiDAR images for each building in preparation for supervised learning and subsequent use in roof shape classification.

Satellite, LiDAR, and building outline data sources have their own spatial reference systems (SRS). The SRS defines a map projection and determines the transformations needed to convert to a different SRS. These reference systems are uniquely identified though a spatial reference system identifier (SRID) which designates an authority and an identifier. For example, the European Petroleum Survey Group (EPSG) can be used to specify SRIDs. Many map vendors, such as OSM, choose to store building outlines as polygons, with each vertex stored in WGS84 (EPSG:4326). Satellite images from common map vendors (ArcGIS, Bing, and Google) often use WGS84/Pseudo-Mercator (EPSG:3857). LiDAR data are usually stored in a region-specific SRS; for example, data for Witten, Germany uses EPSG:5555. To convert a point stored in one SRS to another, a program specialized in these transformations, such as proj.4, must be used [23]. Building polygons are transformed to their LiDAR and satellite counterpart coordinate systems so that the building outlines are consistent.

### 3.2. LiDAR Image Construction

A depth image representation of each building’s roof shape is generated from a LiDAR point cloud. However, many outlier points can inadvertently be present during image generation leading to poor quality or misleading images. To attenuate these effects, bulk preprocessing and per-building filtering steps are performed as described below.

#### 3.2.1. Bulk Preprocessing

LiDAR point cloud data are often stored and publicly released in an industry-standard LASer file binary format [24]. This specification not only details the storage of the xyz coordinates of each point, but also supports data classification. If the LAS file’s ground points have been classified previously, one can filter the ground points from the file leading to improve image generation. However, if the ground points are not already classified, ground point removal per building can be performed as outlined in Section 3.2.2.

Airborne LiDAR point clouds often include points from building wall surfaces that are not of interest for roof shape classification. These points appear as noise around the edges of the generated LiDAR image and can be removed by estimating the normal vectors for each 3D point and removing points that are nearly orthogonal to the unit vector $\hat{k}$ facing up. Normal vectors may be estimated by gathering points in a configurable search radius, *r*, and then performing a least squares fit to a plane. The authors chose to use the open source *White Box Analysis Tools* for generating normal vectors in bulk [25]. A search radius of one meter was chosen to generate a point normal, $\hat{n_{i}}$ for each point $\hat{p_{i}}$, with points stored that satisfy $|\hat{n_{i}}\cdot \hat{k}|>0.3$. This ensures that only points with normals that are within 72° of $\pm \hat{k}$ are kept for further use.

#### 3.2.2. Individual Building Filtering and Projection

Individual building LiDAR filtering begins by constructing a 2D planar bounding box (BBOX) from a polygon building outline. This BBOX is used first to quickly remove points in the point cloud that are not related to the building of interest. The resulting subset of points is filtered again using the polygon roof outline, resulting in only points encapsulated in the building outline. Points are determined to be within the polygon by employing a ray casting algorithm [26]. At this time, the 3D point cloud may be noisy and contain undesirable points.

Ground points not already removed due to a ground label per Section 3.2.1 must now be removed. First, the minimum ground height $z_{min}$ must be identified; this value is specific to the building of interest. Ground height can be determined by applying a buffer to the BBOX ensuring a ground point is within the set and then finding the point with the minimum height. Any point whose z coordinate, ${\hat{p}}_{i,z}$, less than $z_{min}$ plus a configurable threshold $z_{buff}$ can be considered a ground point and then removed, as shown in Equation (1). The authors found $z_{buff}=2.5$ meters is sufficient to remove most ground points. Note this fractional $z_{buff}$ accounts for sheds, etc. with low height.

(1) $$z_{min}+z_{buff}<{\hat{p}}_{i,z}$$

**Figure 4:**
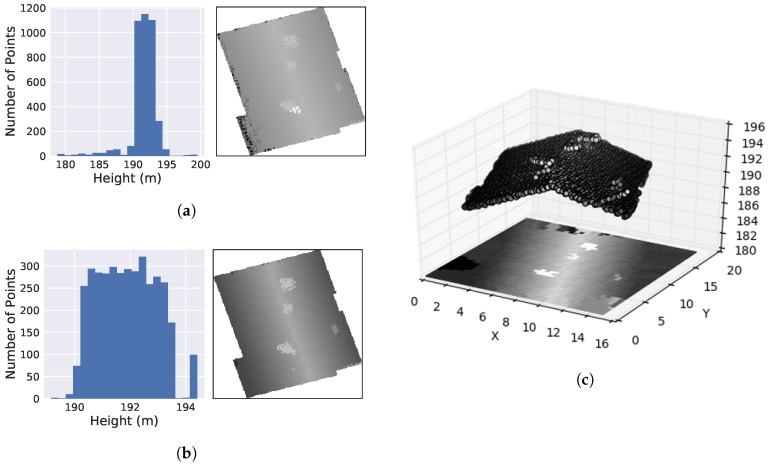
LiDAR data of a gabled roof. Histogram of height distribution and generated image (**a**) before filtering and (**b**) after filtering, using median absolute deviation. (**c**) Projection of filtered point cloud.Results of LiDAR Filtering

A final step of filtering will remove stray points often caused by overhanging trees or other interference. This technique relies upon analyzing the distribution of the *z*-coordinates of each building’s point cloud. This paper employs median absolute deviation (MAD) to construct a modified z-score that measures how deviant each point is from the MAD as in [27]. This method only applies to unimodal distributions; however not all buildings height are distributed as such. For example, there exist complex flat buildings that contain multiple height levels resulting in a multimodal distribution. To distinguish these buildings, the dip test statistic is employed which measures multi-modality in a sample distribution [28]. The test outputs a p-value ranging from zero to one, with values 0.10 or less suggesting bimodality with marginal significance [29]. Any building with a p-value greater than 0.2 is considered unimodal, and outlier removal is performed as shown in Algorithm 1. Results of this filtering technique are shown in Figure 4.

**Algorithm 1:** Filtering of 3D LiDAR point cloud using Medium Absolute Deviation

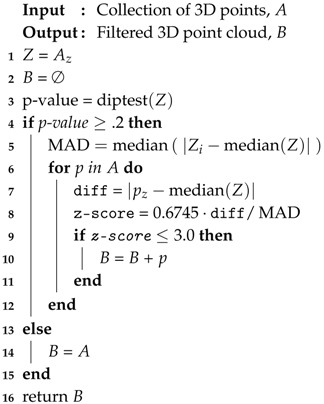



Once LiDAR point extraction is complete, the points are projected onto a plane, creating a 2D grid that takes the value of each point’s height information. The 2D grid world dimensions are the same as the bounding box of the building, with the discrete grid size being the desired square image resolution. Grid points use interpolation of nearest neighbor if no point is available. Afterward, this grid is converted into a grayscale image, where each value is scaled from 0 to 255 with higher values appearing whiter and lower areas darker. Figure 4c demonstrates this process. The CNN’s used in this paper require the grayscale LiDAR data be converted to a three-channel RGB image by duplicating the single channel across all three color channels. This final image is referred to as the LiDAR image.

### 3.3. Satellite Image Construction

**Figure 5:**
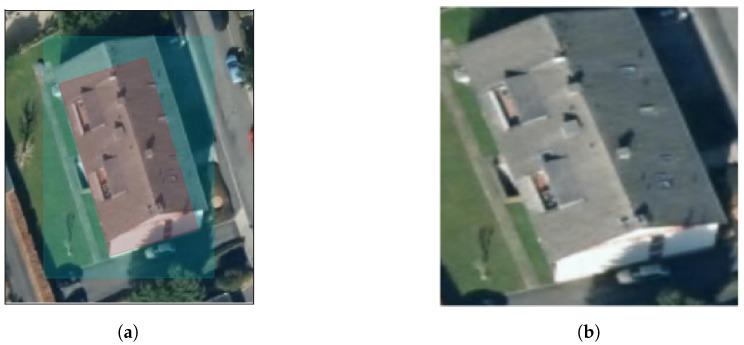
(**a**) Witten building outline in red shading overlaid on the satellite image. The enlarged crop area is shown in cyan shading. (**b**) The final generated image, resized.

It is preferable that the satellite imagery be orthorectified to remove image tilt and relief effects. Ideally, the building polygon can be used to completely stamp out a roof shape image. However, if the aforementioned issues are present in the image, it is unlikely that the polygon will exactly match the building outline in the image. To work around these issues, an enlarged crop can be made around the building. The enlarged crop is produced by generating a buffer around the building polygon by a configurable constant, and then using the bounding box of the new polygon as the identifying stamp. After the image is produced, the image is resized to the square image resolution required by the CNN. The authors found this technique to be necessary only in Witten, while Manhattan and Ann Arbor building outlines were fairly consistent with satellite images. After experimentation, this configurable constant was set to three meters when processing the Witten dataset. Figure 5a shows an example original building outline (red shade) overlaid on a satellite image, and the expanded polygon bounding box in cyan. The resulting generated image is shown in Figure 5b. This final image is referred to as the RGB image below.

### 3.4. Stage 1: CNN Architectures and Training

**Figure 6:**
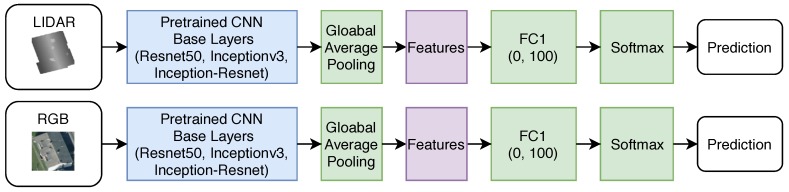
CNN architecture templates.

The CNN base architectures chosen for experimentation are Resnet50 [30], Inceptionv3 [31], and Inception-ResNet [32]. All three of these architecture structures are distinct; when trained and tested on ImageNet [17] they received “Top 5” accuracy scores of 92.8%, 93.9%, and 95.3%, respectively. The computational complexity and size of the network increases progressively from Resnet50 to Inceptionv3, with the Inception-ResNet architecture combining the previous architectures to produce a deeper overall network. Each CNN makes use of successive convolutional blocks to generate a final feature map (referred to as the base layers) which are subsequently used by downstream fully-connected layers to make a 1000 categorical prediction (referred to as the top layers). The top layers are domain specific and are not needed for roof classification thus are removed. This paper applies a global average pooling layer after the final feature layer of each architecture, reducing the convolved feature layers to be used as input into a roof classifying layer. This final classifying layer is composed of an optional fully connected layer (FC1) and a softmax prediction layer as shown in Figure 6. A FC1 size of 0 means the fully connected layer is omitted, and the features map directly to the softmax layer. These models are then trained individually on the RGB and LiDAR images.

Training initializes base layer weights with their respective parent architecture. The optimizer chosen for gradient descent is Adam [33] for its ability to effectively adjust learning rate automatically for individual weights; this optimizer is kept consistent for all architectures and training sessions with learning rate initialized at 0.001. The option of freezing initial layers is exploited with a variable number of frozen layers chosen. When Layer 11 is said to be frozen, this means all previous layers, (Layers 1–11), are frozen during training. All base architectures and tested hyperparameters are shown in Table 1.

Keras [34], a high-level neural network API written in Python, is used to import the pretrained CNN models and construct the new architectures discussed above. A maximum of 1000 epochs are run during the training process, while early stopping is employed at the end of each epoch. Early stopping is a technique where after each epoch, the model is run against the validation set and accuracy metrics are reported. If validation accuracy is not improved after seven epochs, training is halted. This ensures that the the model does not needlessly overfit the training data, and the most generalized model is saved. Data augmentation is performed randomly with horizontal and vertical image flips as well as rotations ranging from $0^{{}^{\circ}}$–$45^{{}^{\circ}}$.

After training is complete on all CNN architectures and hyperparameters, the best performing CNN with respect to the validation set accuracy for both LiDAR and RGB images is selected for further use. Another training session is performed to determine if region-specific training improves region model accuracy, i.e., whether a model that is trained with data in a specific region (city) will be better at predicting roof shapes in that region compared to a model trained on more diverse data. In this study, model architecture is held constant; only training data quantity and diversity are manipulated.

### 3.5. Stage 2: SVM and Random Forest Classifier Training

**Figure 7:**
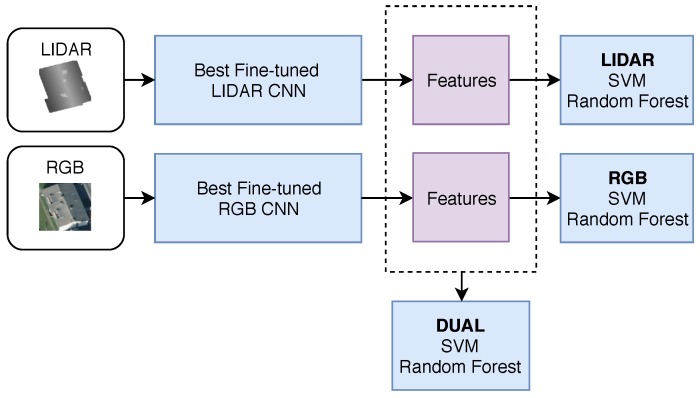
Feature extraction for use in SVM and Random Forest model training. The “dual” model refers to both LiDAR and RGB features being combined as input for model training and prediction.

The best CNN models are used to extract high level image features as input to a downstream “Stage 2” classifier. This step determines if improved results can be obtained by combining both classical and deep learning models together, as shown in Figure 7. In this scenario, only the layers up to global average pooling are used to generate a condensed feature map for each image in the dataset. The augmented training set images are *reduced* to this small feature vector and are used to train both sets of classifiers (SVM and random forest) over a variety of configurations, as shown in Table 2. The Python machine learning library Scikit-learn is used to train and validate the models [35]. The final model is chosen which holds the highest test score accuracy.

## 4. Results

### 4.1. Case Study Dataset Generation

This section outlines the data sources of several cities used to generate images of building rooftops for this paper’s case studies. Procedures for manually labeling images are discussed, and a complete breakdown of labeled roof categories is presented. Example images are shown for each category along with explanations of training, validation, and testing datasets.

#### 4.1.1. Data Sources

The geographic regions used in the following case studies are chosen to maximize diversity in roof shape architectural examples. Diversity within each class translates to image differences such as colors and outline shapes for roofs. Data from the cities of Witten, Germany; the Manhattan borough of New York City, New York; and Ann Arbor, Michigan are used to generate case study data. Witten represents a small urban city with minimal high rise buildings and numerous single-family residential buildings, whereas Manhattan represents a sprawling metropolis with a diverse range of flat-like building roofs with structural additions to the rooftops (antennas, water towers, air conditioning units, etc.). Ann Arbor, used only as an independent test set, includes a combination of building architectures found in Witten and Manhattan. Each of these cities provide publicly available high resolution satellite images, LiDAR data, and building outlines per Table 3. Building sampling was random in the downtown districts of Ann Arbor and Manhattan, while Witten was sampled uniformly over the entire city.

#### 4.1.2. Image Generation and Labeling

**Figure 8:**
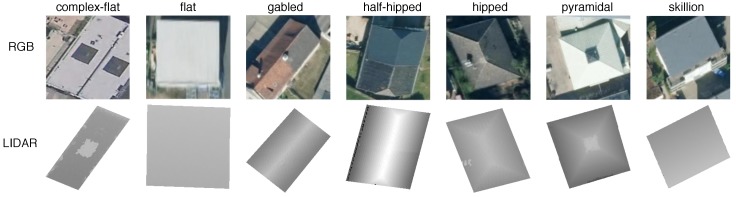
RGB and LiDAR example images of roof shapes.

**Figure 9:**
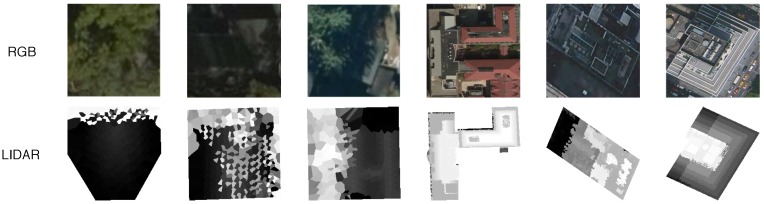
RGB and LiDAR example images classified as unknown. This category includes buildings with poor quality images as well as complex roof structures.

Using the methods described in Section 3, RGB and LiDAR images are generated for each building roof in all cities and then randomly downsampled. All data are treated as unlabeled, requiring manual classification by the authors. One of eight roof shape labels can be assigned to each image: unknown, complex-flat, flat, gabled, half-hipped, hipped, pyramidal, and skillion (shed). This set was determined by observing the most abundant roof architectures present in Witten and Manhattan and merging them together. Unknown is a catch-all category used to account for roof shapes outside the other seven, often labeled complex in other literature [12,15]. Additionally, poor quality images unsuitable for roof prediction are also marked unknown. A complex-flat roof differs from a flat roof in the significance of obstructions on the surface of the roof, or if there are multiple height layers. A flat roof should have minimal objects and a near homogeneous height profile, while a complex-flat roof may have additional items such as water towers or superstructures but still contain sufficient flat structure, e.g., for a safe small UAS landing. This distinction is more apparent in Manhattan than Witten; separating these categories is beneficial to provide class diversity in an otherwise architecturally binary dataset. Practically all roofs in Manhattan are either flat-like or classified as unknown. Examples of RGB and LiDAR images for the seven classes of roof shapes are shown in Figure 8 while examples of the unknown class are found in Figure 9.

LiDAR and satellite images may in some cases be labeled differently. For example, a building with an actual gabled roof may have a LiDAR image which is malformed leading to an unknown class label, while the RGB image may be clear leading to a gabled label. These differences must be noted to prevent models from being trained on incorrect classifications; we want the LiDAR model to learn that the LiDAR image is poor and that an unknown classification should be given while the RGB model should learn the true label. When label differences occur, both labels are kept for training and model validation, leading to *differences* between the LiDAR and RGB **training** and **validation** datasets. However, the **test** dataset *do not* have these label difference between modalities; the test set instead marks every image with the true building label. This ensures that the test set presents an accurate prediction of results with slightly lower classifier accuracy than validation datasets. If both modality images are poor, then the true label is unknown because no prediction is possible.

A final rare case exists where one modality is clear and correctly labeled but the other modality is *misleading* with an incorrect label. This occurs especially in LiDAR images of half-hipped buildings appearing as though they are gabled. There is often only a subtle difference between the two classes, a small triangular dip near the edge of the building, that may not be captured fully in the LiDAR image. When this occurs, the LiDAR image is removed from the training/validation set because one does not want to train on an image that will give inaccurate results. However, the test dataset is left intact. In all cases, the test dataset holds the true roof label based on manual classification, and performance of all machine learning models is assessed in comparison to predicting the true label.

Models that require both input modalities for prediction must have a single label reference for training. If a conflict exists between the two image labels, then the true label is used as was done in the test dataset. This is beneficial as it forces the model to learn to rely on another modality when one input is known to be incorrect. A complete breakdown of the annotated dataset by city is in Table 4. Witten and Manhattan data are combined together and divided into training, validation, and testing data in a 60/20/20 random split. The Ann Arbor data are used only as a secondary test set to determine generalizability of the model and results.

Note that some data were removed from each city because of discrepancies between satellite and LiDAR data resulting from the time the data were recorded. For example, a newly constructed neighborhood in Witten has newer satellite images capturing the neighborhood while old LiDAR data show a flat undeveloped area. This situation was attenuated in Manhattan by looking at building construction dates and only using buildings whose date of construction is before the creation of earliest data sources. However, this information was not able to be found for Witten leading to a much higher removal rate. Overall, about 5.6% of the data were manually discarded for Dataset 1 (Witten and Manhattan). No buildings were removed from the Ann Arbor dataset used for testing.

### 4.2. CNN Training and Results

**Figure 10:**
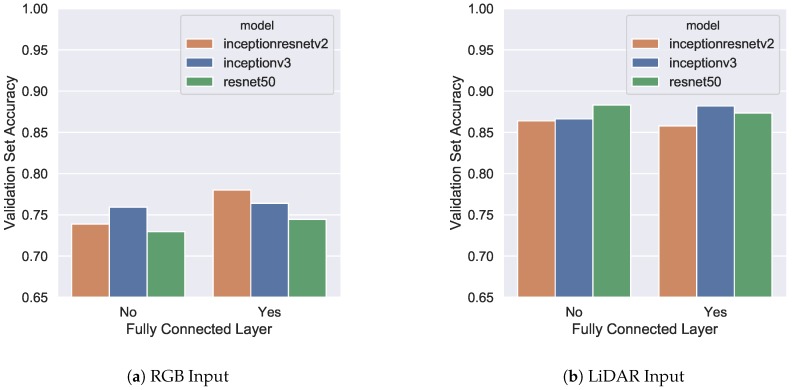
Comparison between CNN validation set accuracy. Colors indicate the base model used while the horizontal axis specifies whether a fully connected layer is part of the architecture. (**a**) RGB (satellite) image input and (**b**) LiDAR image input.

All training was performed on the University of Michigan Flux system, providing a server with a minimum of six gigabytes of RAM, two CPU cores, and a single NVIDIA Tesla K40. The training and validation was performed only on Dataset 1, the combination of the Manhattan and Witten data. Figure 10a plots validation set accuracy for the best-performing CNN models with RGB input, while Figure 10b displays results for LiDAR input. The horizontal axis of both figures indicates whether the network uses a fully connected layer after features are extracted from each CNN. Consistent with previous research, accuracy results are substantially higher (∼10%) using LiDAR data versus RGB data. The best performing network for RGB input is Inception-Resnet with a fully connected layer providing a validation set accuracy of 78.0%. Accuracy appears to increase for RGB input models with increasing CNN model complexity as well as the addition of a fully connected layer.

The best performing model for LiDAR input was Resnet50 with a validation set accuracy of 88.3%, which narrowly outpaced Inceptionv3 with a score of 88.1%. The accuracy differences are statistically insignificant, however the difference in model complexity in terms of memory and computations is significant. Resnet50 is approximately 50% smaller in amount of floating-point operations and took 36 min to train versus the 81 min Inceptionv3 required [43]. In fact, all models performed similarly, and the addition of a fully connected layer (adding more complexity) provided marginal benefit for accuracy. All these factors indicate that a simpler model is desirable for LiDAR input. Intuitively, the complex nature of satellite RGB images would necessitate a deeper network to extract useful features, while the more simplistic LiDAR images would require a less complicated model. The final model architectures chosen are displayed in Table 5 along with their training parameters.

**Figure 11:**
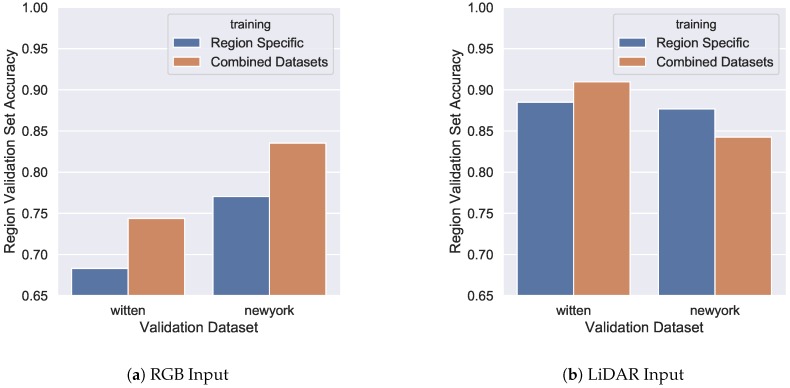
Comparison between region-specific and combined training datasets. Results labeled “combined dataset” are trained on images from both Witten and Manhattan. Validation set accuracy on the vertical axis is specific to the region indicated on the horizontal axis. (**a**) RGB (satellite) image input and (**b**) LiDAR image input.

Using the best performing models, as shown in Table 5, another region-specific training session was performed. Concretely, the training and validation datasets are separated by region, one for Witten and one for Manhattan (New York), and the same architectures are retrained on this subset of the original combined data. Figure 11 shows the results of comparing these new region-specific models to the previous combined models. Accuracy results are significantly higher for RGB input by using the model trained on the combined dataset, clearly demonstrating the benefits of data quantity and diversity. However, LiDAR input has mixed results, with Witten performing better with additional data and Manhattan performing worse. It is possible that the limited amount of class diversity in the Manhattan dataset has not benefited by the diverse architectural examples Witten provides. However, the results as a whole indicate that the models trained on the combined dataset are overall more accurate and should be chosen for use in new cities.

### 4.3. Feature Extraction for SVM and Random Forest Training

**Figure 12:**
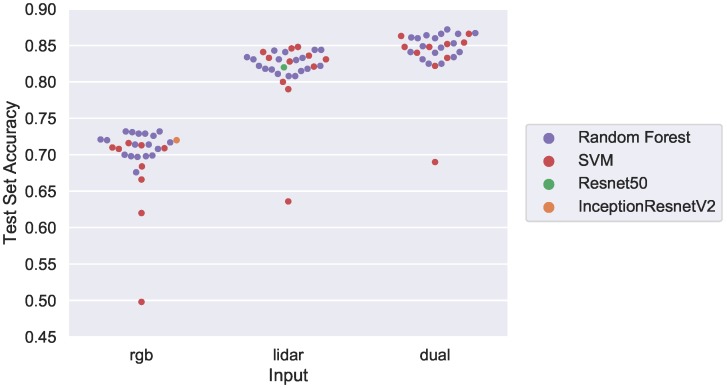
Test Set 1 (Witten/Manhattan) accuracy using CNN feature extraction coupled with SVM and decision tree classifiers.

Training set images from Manhattan and Witten have their salient features extracted using the trained models in Table 5. These features come after the global average pooling layer and are vector sizes of 1536 and 2048 for Inception-Resnet and Resnet, respectively. This new high level feature training set is then fed to SVM and random forest classifiers with varied configurations for training as specified previously in Table 2. Once all classifiers are trained, they are run against Test Set 1 (Witten and Manhattan). Results are shown in the Figure 12 swarm plot where each dot represents a model trained at a different configuration; input modality is determined by its placement on the horizontal axis. The color represents base model type, and CNN accuracies are also shown for comparison. The y-axis is configured to begin at 45% accuracy, truncating low accuracy outlier models. There are six outliers *not* shown which are all SVM models using a polynomial kernel.

As before we see an increase in model accuracy using LiDAR data in comparison to only RGB, and even higher accuracy is achieved by combining the features into a “dual” input classifier. Focusing on RGB input, the best classifiers are all random forest, with the top classifier achieving 73.3% accuracy. This result scores higher than CNN accuracy, underscoring the strengths of random forests for generalized classification. In this instance, the random forest was configured with 50 maximum estimators, an entropy split, and a maximum depth of 10.

LiDAR models score significantly higher, with both SVM and random forest models achieving similar top accuracies of 84.8% versus 84.4%, respectively. This top scoring SVM is configured to use a radial basis function (rbf) kernel with a regularization constant of 10, while the random forest is the same configuration that scored highest for RGB input. Once again, these classical machine learning algorithms outperformed the CNN network in classification on the reduced feature set.

The dual input results validate previous research in that combining multiple streams of modality data can lead to greater accuracy than use of either data type individually. The top classifier is once again a random forest with the same configuration previously discussed; this configuration performs consistently well in all classification tasks. Overall, an improvement of 2.4% is observed by fusing features together resulting in an accuracy of 87.2%. Table 6 shows the top model classifiers and associated parameters for each modality. The authors chose the “dual” input random forest classifier for the final analyses described below.

Three SVM model outliers can be seen for all three inputs. The RGB outlier model used a sigmoid kernel, while the LiDAR and dual input model outliers used a polynomial kernel. No random forest model provided a low test accuracy to be considered an outlier.

### 4.4. Analysis of Final Dual Input Model

Section 4.4.1 provides analysis for the final dual input random forest model by generating confusion matrices for both Test Set 1 and Test Set 2. Section 4.4.2 aggregates flat-like classes for the UAS emergency landing application and evaluates the tradeoff between precision and recall through confidence thresholding.

#### 4.4.1. Confusion Matrices

**Figure 13:**
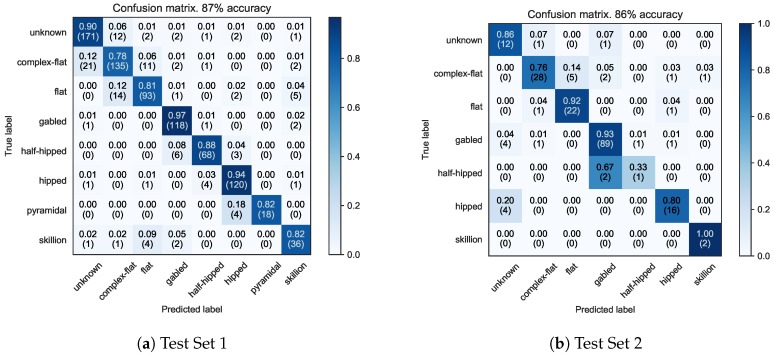
Confusion Matrices for Test Set 1 (Witten/Manhattan) and Test set 2 (Ann Arbor).

The total accuracy for Test Set 1 (Witten and Manhattan) is 87.2%, while Test Set 2 (Ann Arbor) scored 86.7%. The final dual input model’s confusion matrices for Test Set 1 and Test Set 2 are shown in Figure 13a,b, respectively. The row-wise percentage of each cell is computed and color coded along with the specific quantity classified in parentheses underneath. We can see that for both test sets one of the largest errors comes from the confusion between complex-flat and flat roofs. The authors found difficulty in labeling some flat-like roof examples, especially ones that bear traits of both classes; it is clear this confusion carried over into the trained model. In some cases, a roof is on the threshold of being flat or complex-flat, and this ambiguity makes it difficult to provide a consistent “correct” answer. Indeed, this case often applies between the complex-flat and unknown labels as well: When does a complex-flat roof become too complex to support a safe small UAS landing? The authors attempted to be consistent in answering this question when labeling data, however edge cases were observed. Table 7 and Table 8 list results for recall (completeness), precision (correctness), and quality for Test Set 1 and Test Set 2, respectively. Note that there were no pyramidal roofs shapes in the Ann Arbor test set and too few half-hipped and skillion roofs to calculate valid metric results.

#### 4.4.2. Confidence Thresholding

**Figure 14:**
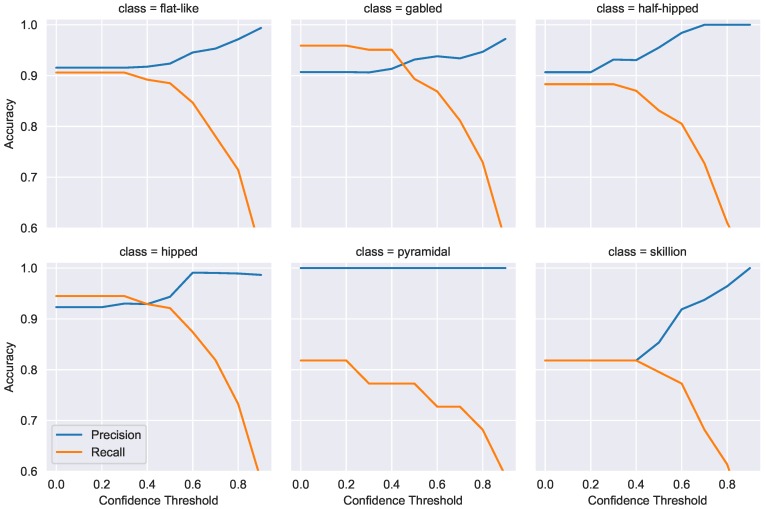
Test Set 1 confidence threshold impact on precision and recall for multiple classes.

**Figure 15:**
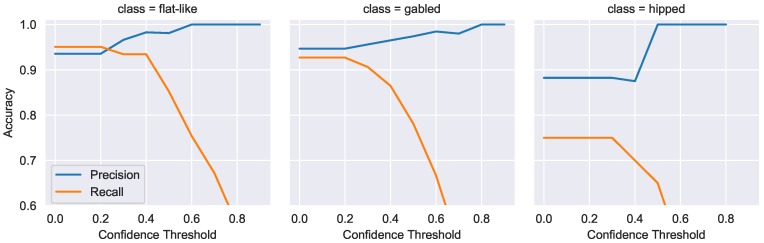
Test Set 2 confidence threshold impact on precision and recall for multiple classes.

With every model prediction, there is a probability distribution of the likelihood the example belongs to a class. The class with the highest probability is then chosen as the final prediction. Model precision can be increased by adjusting the confidence threshold a model requires to make a prediction, and, if not met, the example is marked unknown. This will generally decrease the number of false positives at the expense of an increase in false negatives. For the UAS emergency landing use case, operators need confidence that a roof labeled as “flat-like” is actually flat. We use confidence thresholding to combine complex-flat and flat roofs into one flat-like category used for UAS roof identification. Figure 14 shows individual graphs of how the model’s predictive power on Test Set 1 is impacted as the confidence threshold is manipulated. This process is repeated on Test Set 2 in Figure 15, with half-hipped, skillion, and pyramdial classes omitted due to lack of examples.

We can clearly see the inverse relationship between precision and recall as the required confidence threshold is increased. Unfortunately, this relationship is clearly not linear for all classes; moderate increases in precision come at a large decrease in recall for flat-like, gabled, half-hipped, and hipped classes. Indeed, as precision increases above 95% recall drops exponentially to around 60%. These figures certainly show there is a limit to the effectiveness of confidence thresholding; setting too high a confidence threshold may even lead to a *drop* in precision in some cases as seen for the hipped class in Figure 14. However, these results show promise that class-specific thresholds can be set to ensure high-precision predictions are generated. For UAS landing, these results indicate we can achieve near-perfect precision at the expense of only finding ∼60% of the flat roofs within a region.

## 5. Discussion and Future Work

The presented study demonstrates that a combination of a Stage 1 CNN feature extractor coupled with a Stage 2 random forest classifier can reliably and effectively label city wide roof shapes with publicly available GIS data. In addition, we show good generalization of our final model on diverse city landscapes ranging from small to large urban centers. Two independent test sets show similar results in model quality metrics providing a realistic expectation of model performance, where one set, Ann Arbor, was not used in training. Others have successfully performed roof shape classification through machine learning, but no previous work to-date has demonstrated effectiveness to the scale analyzed here in both breadth and depth. Over 9000 images (two for each building) have been manually labeled from three diverse cities to generate the training, validation, and test sets. In comparison, the largest labeled dataset the authors found in the literature for roof top classification is 3000 images and encompasses only one city [16].

A comparison of our accuracy results with other work is difficult because no benchmark test set has been available to date for roof shape classification. Benchmarking datasets are of critical importance to compare the results of applying different algorithms [44]. Since no such benchmarking data exist for roof shape classification, the authors propose this paper’s released annotated dataset serve as an initial dataset for future roof shape classification research.

The challenge of comparing algorithms is compounded by differences in expected model input. Many models preprocess LiDAR input into handcrafted features, such as slope, aspect, and number of roof surfaces [12,13]. Others rely on a raw DSM image of a roof, while our work relies upon automatically generating a depth image from point clouds specifically filtered for each building roof. Our work is one of the few that relies upon both satellite images and LiDAR data for classification, and is the only one that uses deep learning to train on both modalities together to enhance model accuracy. In addition, our work classifies eight roof categories, naturally bringing down accuracy results in comparison to most others works attempting to classify six or at most seven roof shapes.

The largest weakness in this study comes from one of its greatest strengths: the fusion of LiDAR and satellite input is only effective if both data sources observe the same thing. If one modality sees a newly constructed neighborhood and the other sees undeveloped area, for example, the model will become confused. The authors attempted to mitigate this issue by looking at construction dates for buildings, and removing buildings constructed during/after the earliest data source. However, this construction information is difficult to obtain in all cities/countries, and does not guarantee the removal of all possible data source inconsistencies. Future work is needed to automatically detect inconsistent datasets if present and automatically label the roof as unknown. Note that inconsistent datasets are immediately apparent to the human eye.

As the authors have continually refined the LiDAR pre/post-processing methods for depth image generation, they have concluded that an alternative method may be more suitable. Instead of painstakingly converting point clouds to high quality depth images for a CNN, it should theoretically be better to operate directly on the point cloud itself in a deep learning model. Several advances have been proposed in deep learning for both point cloud segmentation and classification, e.g., PointNet and SpiderCNN [45,46,47]. These neural network architectures sample from the point cloud and directly learn global and local geometric features of the point cloud surface. These methods have been shown to be successful in small scale object classification (household items, pedestrians, etc.) using high resolution LiDAR data; future work should investigate their use on airborne LiDAR data.

Small UAS rooftop landing requires a high degree of confidence that a flat-like surface exists for safe landing. This paper demonstrates that flat-like roofs can be be reliably predicted with high precision by adjusting the final model’s confidence threshold. After flat-like roofs have been identified, further post processing may be performed to quantify metrics such as ideal landing position, surface roughness, and rooftop geometry. The output of this future work can then reliably generate a database of emergency landing sites that is risk-aware.

## 6. Conclusions

Building outline and height information is useful for visualization and 3D reconstruction but roof shape is often missing or at best incomplete in existing databases. GIS data such as satellite images, LiDAR point clouds, and building outlines are often available. This paper processes these data to construct individual image representations of depth and color of roof shapes. Datasets are constructed and manually labeled across multiple cities. The final model uses deep learning for feature extraction and a random forest algorithm for subsequent roof shape classification. Two test sets from diverse cities show good generalization of the trained model, reporting total accuracies near 87%. Confidence thresholds are manipulated leading to greater than 98% precision in labeling flat-like roofs in all three tested cities, an important increase in precision for applications such as UAS rooftop landing. The generalized models and test datasets show promise for applying machine learning to automatically label roof shapes around the world with high confidence.

## Figures and Tables

**Table 1:** CNN architectures and hyperparameters.

Base CNN Model	FC1 Size	Frozen Layers
Resnet50	0, 100	50, 80
Inceptionv3	0, 100	11, 18, 41
Inception-Resnet	0, 100	11, 18, 41

**Table 2:** SVM and random forest training configurations.

Classifier	Parameters
SVM	Regularization Constant (C): 1, 10, 100
	Kernel: linear, rbf, poly, sigmoid
Random Forest	Criterion: gini, entropy
	Number of Estimators: 5, 10, 50
	Max Depth: 5, 10, 50

**Table 3:** Satellite, LiDAR, and building data sources.

City	Satellite		LiDAR		Buildings
	Provider	Resolution	Provider	Spacing	Provider
Witten	Land NRW [36]	0.10 m/px	Open NRW [37]	0.30 m	OSM [3]
New York	NY State [38]	0.15 m/px	USGS [39]	0.70 m	NYC Open Data [40]
Ann Arbor	Bing [41]	0.15 m/px	USGS [42]	0.53 m	OSM [3]

**Table 4:** Breakdown of roof labels by city.

Roof Shape	Witten	Manhattan	Ann Arbor
unknown	133	792	14
complex-flat	125	785	37
flat	454	129	24
gabled	572	7	96
half-hipped	436	0	3
hipped	591	3	20
pyramidal	110	0	0
skillion	189	0	2
Total	2610	1716	196
Removed	212	65	0

**Table 5:** Best CNN model architectures.

Input	Base Model	FC Layer?	Frozen Layers
RGB	Inception-Resnet	Yes	11
LiDAR	Resnet50	No	80

**Table 6:** Best Classifiers using CNN extracted features.

Input	Model	Parameters	Test Set 1 Accuracy
RGB	Random Forest	Criteria: Entropy, # Estimators: 50, Max Depth: 10	73.2%
LiDAR	SVM	Regularization Coefficient: 10, kernel: rbf	84.8%
Dual	Random Forest	Criteria: Entropy, #Estimators: 50, Max Depth: 10	87.2%

**Table 7:** Results for recall, precision, and quality evaluation metrics for Test Set 1.

Type	Recall	Precision	Quality
Unknown	0.90	0.88	0.80
Complex-Flat	0.79	0.83	0.68
Flat	0.81	0.84	0.70
Gabled	0.97	0.90	0.87
Half-Hipped	0.88	0.91	0.81
Hipped	0.95	0.92	0.87
Pyramidal	0.82	1.00	0.82
Skillion	0.82	0.77	0.66

**Table 8:** Results for recall, precision, and quality evaluation metrics for Test Set 2.

Type	Recall	Precision	Quality
Unknown	0.86	0.60	0.55
Complex-Flat	0.76	0.90	0.70
Flat	0.92	0.82	0.76
Gabled	0.93	0.96	0.88
Half-Hipped	N/A	N/A	N/A
Hipped	0.80	0.84	0.70
Pyramidal	N/A	N/A	N/A
Skillion	N/A	N/A	N/A

## Supplementary Materials

Supplementary File 1

## Abbreviations

The following abbreviations are used in this manuscript:

OSM	OpenStreetMaps
LiDAR	Light Detection and Ranging
RGB	Red Green Blue
CNN	Convolutional Neural Network
UAS	Unmanned Aircraft System
SRS	Spatial Reference Systems
SRID	Spatial Reference System Identifier
MDPI	Multidisciplinary Digital Publishing Institute
