# Supplementary figures and images for: Roof Shape Classification from LiDAR and Satellite Image Data Fusion Using Supervised Learning (part 2 of 2)
Source: Sensors (Basel). 2018 Nov 15;18(11):3960. doi: 10.3390/s18113960 (PMC6264004; doi:10.3390/s18113960)

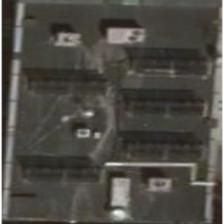

Supplement: Supplementary File 1 [file sensors-18-03960-s001.zip › sensors-377629-supplementary/annarbor/complex_flat/467844846.jpg]

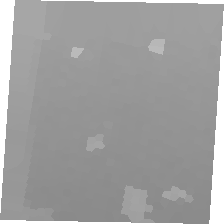

Supplement: Supplementary File 1 [file sensors-18-03960-s001.zip › sensors-377629-supplementary/annarbor/complex_flat/467844846.png]

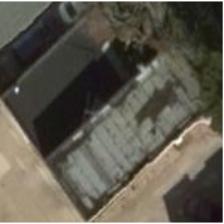

Supplement: Supplementary File 1 [file sensors-18-03960-s001.zip › sensors-377629-supplementary/annarbor/complex_flat/474868753.jpg]

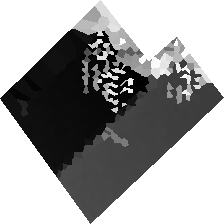

Supplement: Supplementary File 1 [file sensors-18-03960-s001.zip › sensors-377629-supplementary/annarbor/complex_flat/474868753.png]

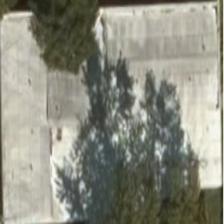

Supplement: Supplementary File 1 [file sensors-18-03960-s001.zip › sensors-377629-supplementary/annarbor/complex_flat/488132674.jpg]

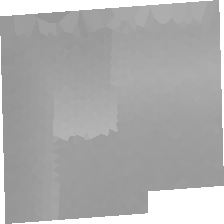

Supplement: Supplementary File 1 [file sensors-18-03960-s001.zip › sensors-377629-supplementary/annarbor/complex_flat/488132674.png]

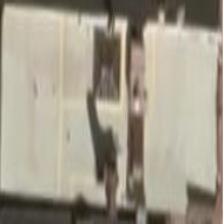

Supplement: Supplementary File 1 [file sensors-18-03960-s001.zip › sensors-377629-supplementary/annarbor/complex_flat/493526519.jpg]

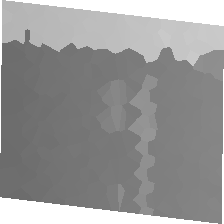

Supplement: Supplementary File 1 [file sensors-18-03960-s001.zip › sensors-377629-supplementary/annarbor/complex_flat/493526519.png]

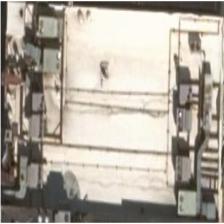

Supplement: Supplementary File 1 [file sensors-18-03960-s001.zip › sensors-377629-supplementary/annarbor/complex_flat/493526520.jpg]

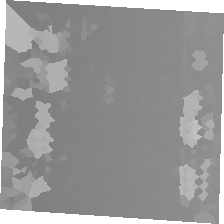

Supplement: Supplementary File 1 [file sensors-18-03960-s001.zip › sensors-377629-supplementary/annarbor/complex_flat/493526520.png]

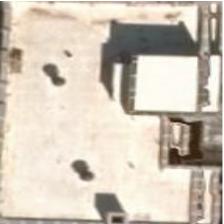

Supplement: Supplementary File 1 [file sensors-18-03960-s001.zip › sensors-377629-supplementary/annarbor/complex_flat/499566248.jpg]

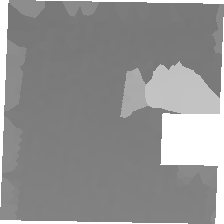

Supplement: Supplementary File 1 [file sensors-18-03960-s001.zip › sensors-377629-supplementary/annarbor/complex_flat/499566248.png]

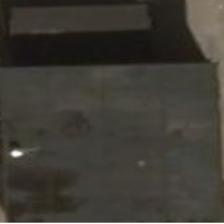

Supplement: Supplementary File 1 [file sensors-18-03960-s001.zip › sensors-377629-supplementary/annarbor/complex_flat/502971264.jpg]

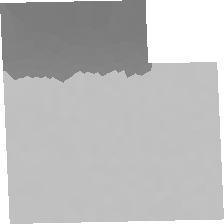

Supplement: Supplementary File 1 [file sensors-18-03960-s001.zip › sensors-377629-supplementary/annarbor/complex_flat/502971264.png]

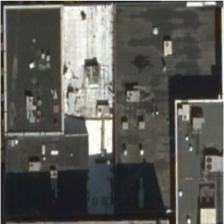

Supplement: Supplementary File 1 [file sensors-18-03960-s001.zip › sensors-377629-supplementary/annarbor/complex_flat/512092372.jpg]

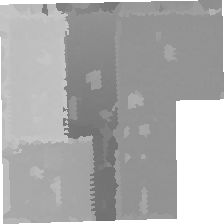

Supplement: Supplementary File 1 [file sensors-18-03960-s001.zip › sensors-377629-supplementary/annarbor/complex_flat/512092372.png]

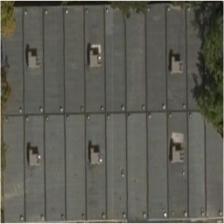

Supplement: Supplementary File 1 [file sensors-18-03960-s001.zip › sensors-377629-supplementary/annarbor/complex_flat/562286287.jpg]

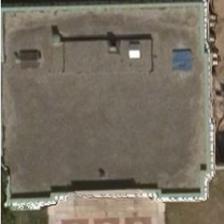

Supplement: Supplementary File 1 [file sensors-18-03960-s001.zip › sensors-377629-supplementary/annarbor/complex_flat/99883671.jpg]

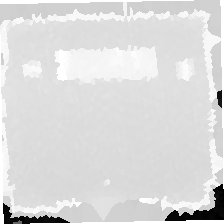

Supplement: Supplementary File 1 [file sensors-18-03960-s001.zip › sensors-377629-supplementary/annarbor/complex_flat/99883671.png]

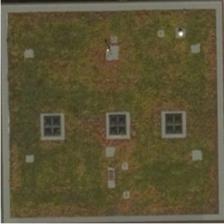

Supplement: Supplementary File 1 [file sensors-18-03960-s001.zip › sensors-377629-supplementary/annarbor/flat/154202674.jpg]

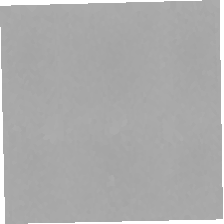

Supplement: Supplementary File 1 [file sensors-18-03960-s001.zip › sensors-377629-supplementary/annarbor/flat/154202674.png]

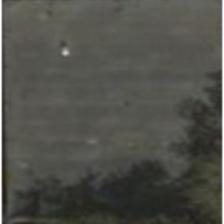

Supplement: Supplementary File 1 [file sensors-18-03960-s001.zip › sensors-377629-supplementary/annarbor/flat/154232140.jpg]

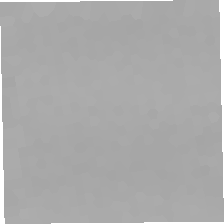

Supplement: Supplementary File 1 [file sensors-18-03960-s001.zip › sensors-377629-supplementary/annarbor/flat/154232140.png]

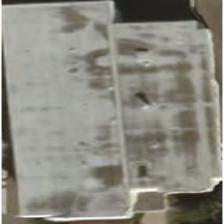

Supplement: Supplementary File 1 [file sensors-18-03960-s001.zip › sensors-377629-supplementary/annarbor/flat/256667241.jpg]

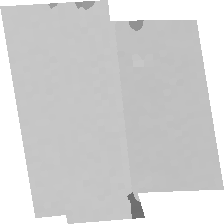

Supplement: Supplementary File 1 [file sensors-18-03960-s001.zip › sensors-377629-supplementary/annarbor/flat/256667241.png]

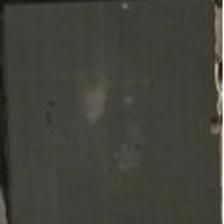

Supplement: Supplementary File 1 [file sensors-18-03960-s001.zip › sensors-377629-supplementary/annarbor/flat/256667297.jpg]

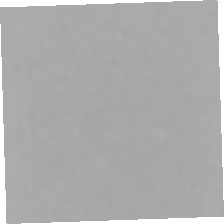

Supplement: Supplementary File 1 [file sensors-18-03960-s001.zip › sensors-377629-supplementary/annarbor/flat/256667297.png]

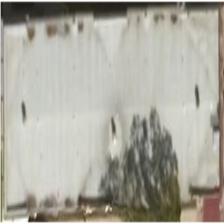

Supplement: Supplementary File 1 [file sensors-18-03960-s001.zip › sensors-377629-supplementary/annarbor/flat/302431407.jpg]

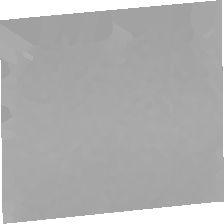

Supplement: Supplementary File 1 [file sensors-18-03960-s001.zip › sensors-377629-supplementary/annarbor/flat/302431407.png]

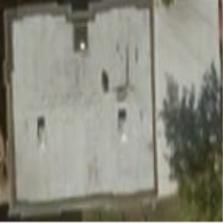

Supplement: Supplementary File 1 [file sensors-18-03960-s001.zip › sensors-377629-supplementary/annarbor/flat/333581755.jpg]

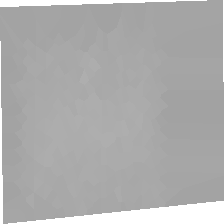

Supplement: Supplementary File 1 [file sensors-18-03960-s001.zip › sensors-377629-supplementary/annarbor/flat/333581755.png]

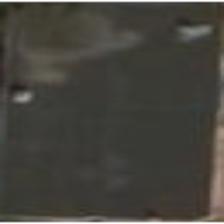

Supplement: Supplementary File 1 [file sensors-18-03960-s001.zip › sensors-377629-supplementary/annarbor/flat/442860969.jpg]

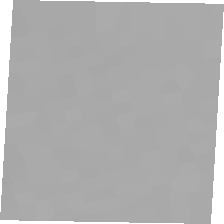

Supplement: Supplementary File 1 [file sensors-18-03960-s001.zip › sensors-377629-supplementary/annarbor/flat/442860969.png]

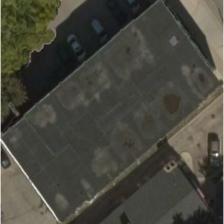

Supplement: Supplementary File 1 [file sensors-18-03960-s001.zip › sensors-377629-supplementary/annarbor/flat/442860982.jpg]

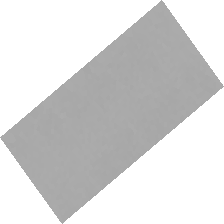

Supplement: Supplementary File 1 [file sensors-18-03960-s001.zip › sensors-377629-supplementary/annarbor/flat/442860982.png]

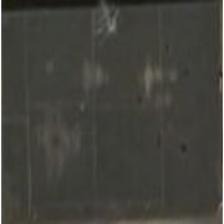

Supplement: Supplementary File 1 [file sensors-18-03960-s001.zip › sensors-377629-supplementary/annarbor/flat/442861010.jpg]

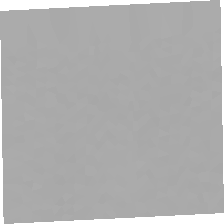

Supplement: Supplementary File 1 [file sensors-18-03960-s001.zip › sensors-377629-supplementary/annarbor/flat/442861010.png]

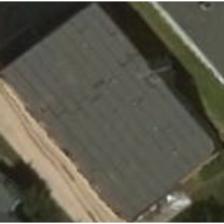

Supplement: Supplementary File 1 [file sensors-18-03960-s001.zip › sensors-377629-supplementary/annarbor/flat/442861011.jpg]

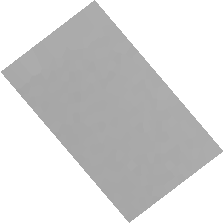

Supplement: Supplementary File 1 [file sensors-18-03960-s001.zip › sensors-377629-supplementary/annarbor/flat/442861011.png]

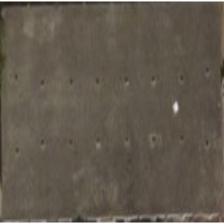

Supplement: Supplementary File 1 [file sensors-18-03960-s001.zip › sensors-377629-supplementary/annarbor/flat/442866611.jpg]

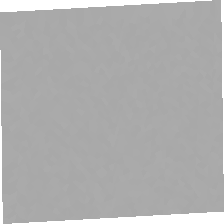

Supplement: Supplementary File 1 [file sensors-18-03960-s001.zip › sensors-377629-supplementary/annarbor/flat/442866611.png]

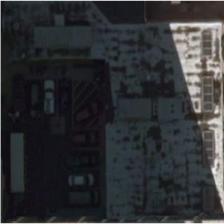

Supplement: Supplementary File 1 [file sensors-18-03960-s001.zip › sensors-377629-supplementary/annarbor/flat/453674959.jpg]

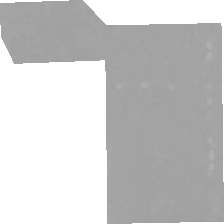

Supplement: Supplementary File 1 [file sensors-18-03960-s001.zip › sensors-377629-supplementary/annarbor/flat/453674959.png]

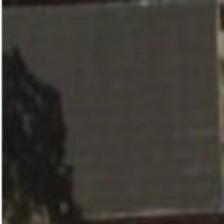

Supplement: Supplementary File 1 [file sensors-18-03960-s001.zip › sensors-377629-supplementary/annarbor/flat/457363243.jpg]

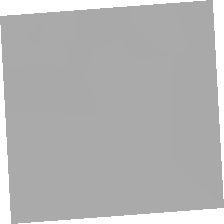

Supplement: Supplementary File 1 [file sensors-18-03960-s001.zip › sensors-377629-supplementary/annarbor/flat/457363243.png]

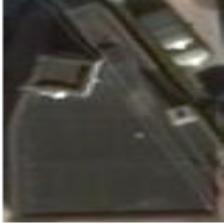

Supplement: Supplementary File 1 [file sensors-18-03960-s001.zip › sensors-377629-supplementary/annarbor/flat/458824133.jpg]

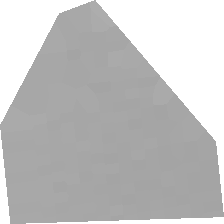

Supplement: Supplementary File 1 [file sensors-18-03960-s001.zip › sensors-377629-supplementary/annarbor/flat/458824133.png]

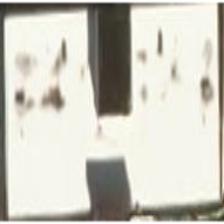

Supplement: Supplementary File 1 [file sensors-18-03960-s001.zip › sensors-377629-supplementary/annarbor/flat/459948088.jpg]

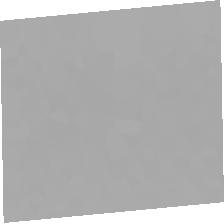

Supplement: Supplementary File 1 [file sensors-18-03960-s001.zip › sensors-377629-supplementary/annarbor/flat/459948088.png]

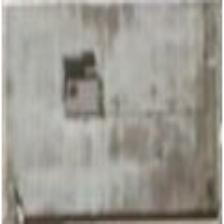

Supplement: Supplementary File 1 [file sensors-18-03960-s001.zip › sensors-377629-supplementary/annarbor/flat/460002075.jpg]

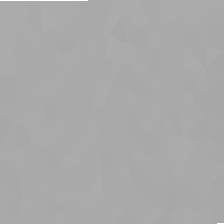

Supplement: Supplementary File 1 [file sensors-18-03960-s001.zip › sensors-377629-supplementary/annarbor/flat/460002075.png]

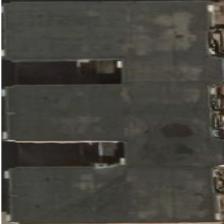

Supplement: Supplementary File 1 [file sensors-18-03960-s001.zip › sensors-377629-supplementary/annarbor/flat/471191917.jpg]

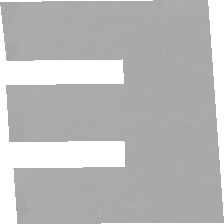

Supplement: Supplementary File 1 [file sensors-18-03960-s001.zip › sensors-377629-supplementary/annarbor/flat/471191917.png]

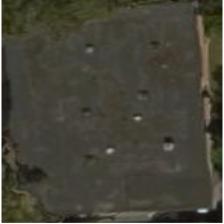

Supplement: Supplementary File 1 [file sensors-18-03960-s001.zip › sensors-377629-supplementary/annarbor/flat/474868755.jpg]

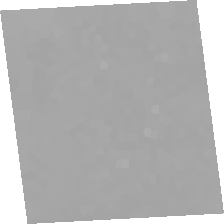

Supplement: Supplementary File 1 [file sensors-18-03960-s001.zip › sensors-377629-supplementary/annarbor/flat/474868755.png]

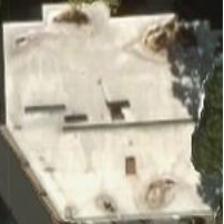

Supplement: Supplementary File 1 [file sensors-18-03960-s001.zip › sensors-377629-supplementary/annarbor/flat/474868756.jpg]

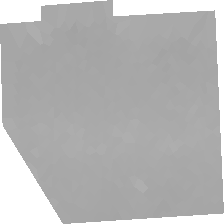

Supplement: Supplementary File 1 [file sensors-18-03960-s001.zip › sensors-377629-supplementary/annarbor/flat/474868756.png]

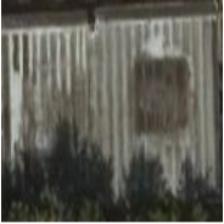

Supplement: Supplementary File 1 [file sensors-18-03960-s001.zip › sensors-377629-supplementary/annarbor/flat/488132670.jpg]

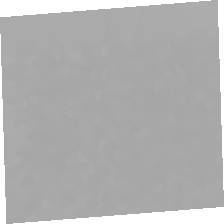

Supplement: Supplementary File 1 [file sensors-18-03960-s001.zip › sensors-377629-supplementary/annarbor/flat/488132670.png]

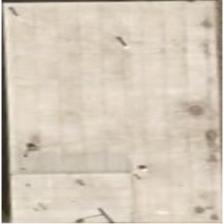

Supplement: Supplementary File 1 [file sensors-18-03960-s001.zip › sensors-377629-supplementary/annarbor/flat/488580464.jpg]

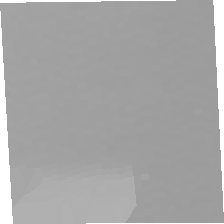

Supplement: Supplementary File 1 [file sensors-18-03960-s001.zip › sensors-377629-supplementary/annarbor/flat/488580464.png]

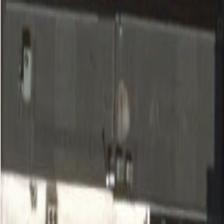

Supplement: Supplementary File 1 [file sensors-18-03960-s001.zip › sensors-377629-supplementary/annarbor/flat/493526528.jpg]

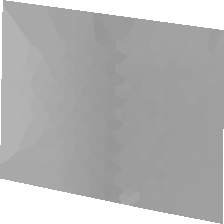

Supplement: Supplementary File 1 [file sensors-18-03960-s001.zip › sensors-377629-supplementary/annarbor/flat/493526528.png]

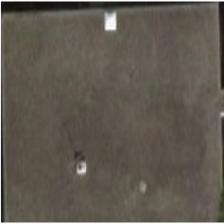

Supplement: Supplementary File 1 [file sensors-18-03960-s001.zip › sensors-377629-supplementary/annarbor/flat/493774993.jpg]

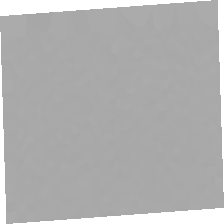

Supplement: Supplementary File 1 [file sensors-18-03960-s001.zip › sensors-377629-supplementary/annarbor/flat/493774993.png]

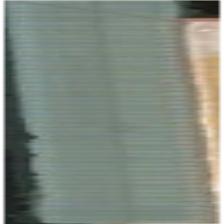

Supplement: Supplementary File 1 [file sensors-18-03960-s001.zip › sensors-377629-supplementary/annarbor/flat/560591414.jpg]

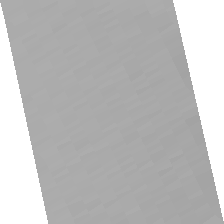

Supplement: Supplementary File 1 [file sensors-18-03960-s001.zip › sensors-377629-supplementary/annarbor/flat/560591414.png]

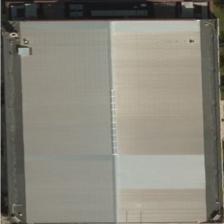

Supplement: Supplementary File 1 [file sensors-18-03960-s001.zip › sensors-377629-supplementary/annarbor/gabled/128634894.jpg]

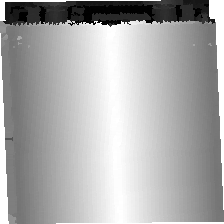

Supplement: Supplementary File 1 [file sensors-18-03960-s001.zip › sensors-377629-supplementary/annarbor/gabled/128634894.png]

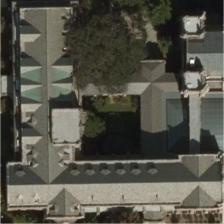

Supplement: Supplementary File 1 [file sensors-18-03960-s001.zip › sensors-377629-supplementary/annarbor/gabled/154203693.jpg]

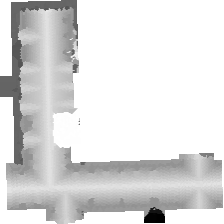

Supplement: Supplementary File 1 [file sensors-18-03960-s001.zip › sensors-377629-supplementary/annarbor/gabled/154203693.png]

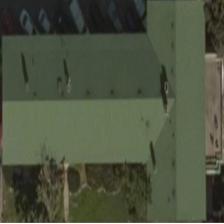

Supplement: Supplementary File 1 [file sensors-18-03960-s001.zip › sensors-377629-supplementary/annarbor/gabled/154203713.jpg]

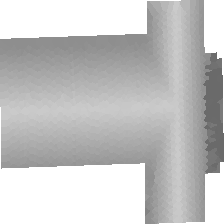

Supplement: Supplementary File 1 [file sensors-18-03960-s001.zip › sensors-377629-supplementary/annarbor/gabled/154203713.png]

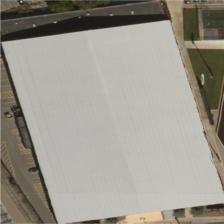

Supplement: Supplementary File 1 [file sensors-18-03960-s001.zip › sensors-377629-supplementary/annarbor/gabled/154232133.jpg]

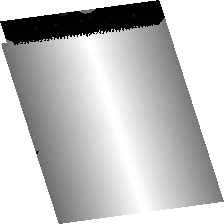

Supplement: Supplementary File 1 [file sensors-18-03960-s001.zip › sensors-377629-supplementary/annarbor/gabled/154232133.png]

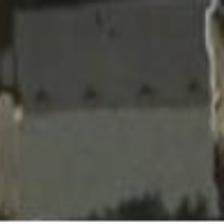

Supplement: Supplementary File 1 [file sensors-18-03960-s001.zip › sensors-377629-supplementary/annarbor/gabled/256667497.jpg]

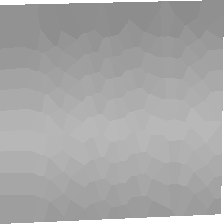

Supplement: Supplementary File 1 [file sensors-18-03960-s001.zip › sensors-377629-supplementary/annarbor/gabled/256667497.png]

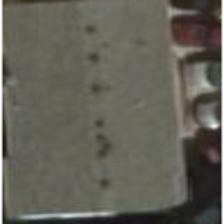

Supplement: Supplementary File 1 [file sensors-18-03960-s001.zip › sensors-377629-supplementary/annarbor/gabled/256668146.jpg]

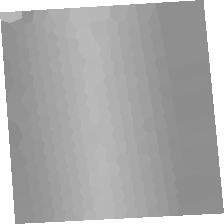

Supplement: Supplementary File 1 [file sensors-18-03960-s001.zip › sensors-377629-supplementary/annarbor/gabled/256668146.png]

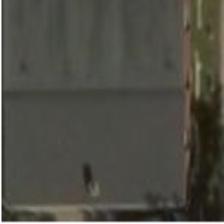

Supplement: Supplementary File 1 [file sensors-18-03960-s001.zip › sensors-377629-supplementary/annarbor/gabled/313630826.jpg]

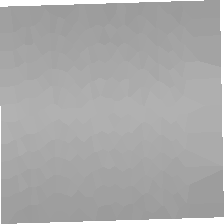

Supplement: Supplementary File 1 [file sensors-18-03960-s001.zip › sensors-377629-supplementary/annarbor/gabled/313630826.png]

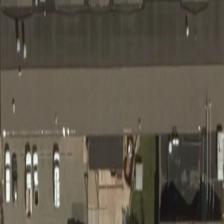

Supplement: Supplementary File 1 [file sensors-18-03960-s001.zip › sensors-377629-supplementary/annarbor/gabled/384454222.jpg]

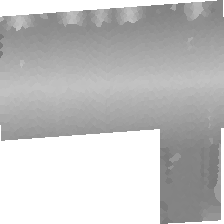

Supplement: Supplementary File 1 [file sensors-18-03960-s001.zip › sensors-377629-supplementary/annarbor/gabled/384454222.png]

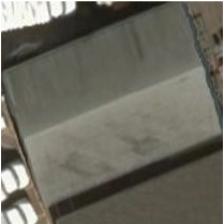

Supplement: Supplementary File 1 [file sensors-18-03960-s001.zip › sensors-377629-supplementary/annarbor/gabled/384455074.jpg]

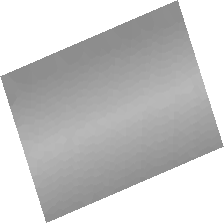

Supplement: Supplementary File 1 [file sensors-18-03960-s001.zip › sensors-377629-supplementary/annarbor/gabled/384455074.png]

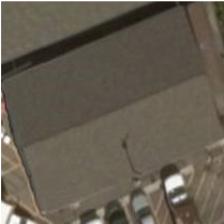

Supplement: Supplementary File 1 [file sensors-18-03960-s001.zip › sensors-377629-supplementary/annarbor/gabled/384455075.jpg]

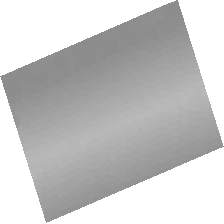

Supplement: Supplementary File 1 [file sensors-18-03960-s001.zip › sensors-377629-supplementary/annarbor/gabled/384455075.png]

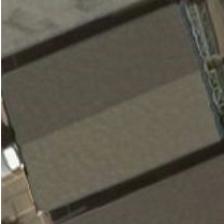

Supplement: Supplementary File 1 [file sensors-18-03960-s001.zip › sensors-377629-supplementary/annarbor/gabled/384455076.jpg]

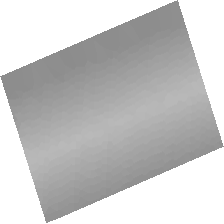

Supplement: Supplementary File 1 [file sensors-18-03960-s001.zip › sensors-377629-supplementary/annarbor/gabled/384455076.png]

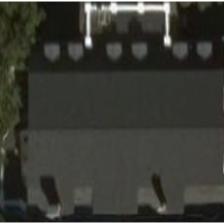

Supplement: Supplementary File 1 [file sensors-18-03960-s001.zip › sensors-377629-supplementary/annarbor/gabled/437310051.jpg]

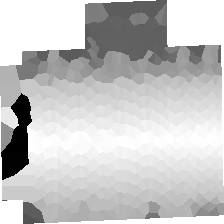

Supplement: Supplementary File 1 [file sensors-18-03960-s001.zip › sensors-377629-supplementary/annarbor/gabled/437310051.png]

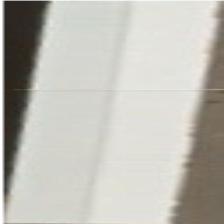

Supplement: Supplementary File 1 [file sensors-18-03960-s001.zip › sensors-377629-supplementary/annarbor/gabled/442861015.jpg]

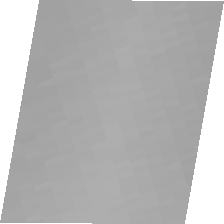

Supplement: Supplementary File 1 [file sensors-18-03960-s001.zip › sensors-377629-supplementary/annarbor/gabled/442861015.png]

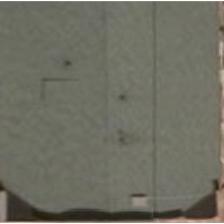

Supplement: Supplementary File 1 [file sensors-18-03960-s001.zip › sensors-377629-supplementary/annarbor/gabled/442864450.jpg]

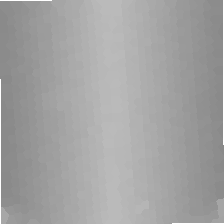

Supplement: Supplementary File 1 [file sensors-18-03960-s001.zip › sensors-377629-supplementary/annarbor/gabled/442864450.png]

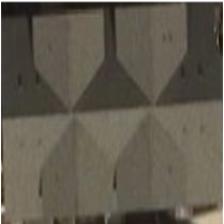

Supplement: Supplementary File 1 [file sensors-18-03960-s001.zip › sensors-377629-supplementary/annarbor/gabled/442866608.jpg]

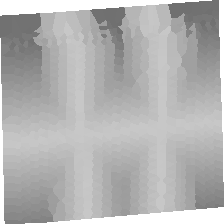

Supplement: Supplementary File 1 [file sensors-18-03960-s001.zip › sensors-377629-supplementary/annarbor/gabled/442866608.png]

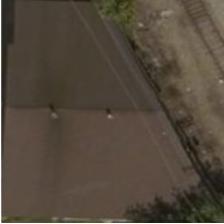

Supplement: Supplementary File 1 [file sensors-18-03960-s001.zip › sensors-377629-supplementary/annarbor/gabled/443277818.jpg]

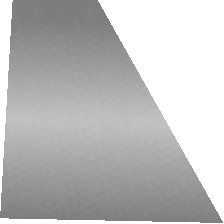

Supplement: Supplementary File 1 [file sensors-18-03960-s001.zip › sensors-377629-supplementary/annarbor/gabled/443277818.png]

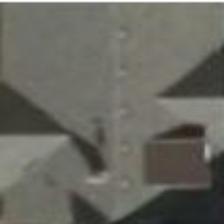

Supplement: Supplementary File 1 [file sensors-18-03960-s001.zip › sensors-377629-supplementary/annarbor/gabled/447333378.jpg]
