# Supplementary figures and images for: Roof Shape Classification from LiDAR and Satellite Image Data Fusion Using Supervised Learning (part 1 of 2)
Source: Sensors (Basel). 2018 Nov 15;18(11):3960. doi: 10.3390/s18113960 (PMC6264004; doi:10.3390/s18113960)

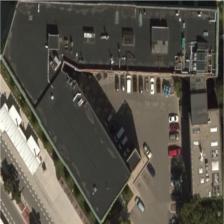

Supplement: Supplementary File 1 [file sensors-18-03960-s001.zip › sensors-377629-supplementary/annarbor/complex/154202605.jpg]

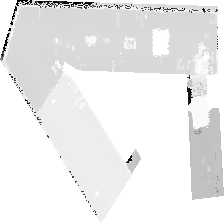

Supplement: Supplementary File 1 [file sensors-18-03960-s001.zip › sensors-377629-supplementary/annarbor/complex/154202605.png]

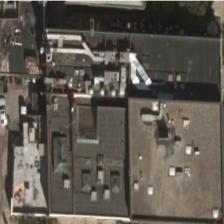

Supplement: Supplementary File 1 [file sensors-18-03960-s001.zip › sensors-377629-supplementary/annarbor/complex/154202623.jpg]

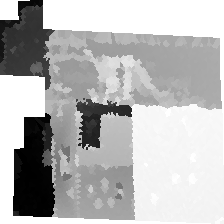

Supplement: Supplementary File 1 [file sensors-18-03960-s001.zip › sensors-377629-supplementary/annarbor/complex/154202623.png]

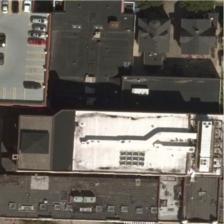

Supplement: Supplementary File 1 [file sensors-18-03960-s001.zip › sensors-377629-supplementary/annarbor/complex/154202627.jpg]

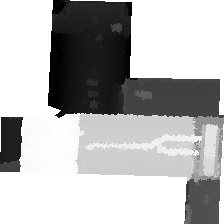

Supplement: Supplementary File 1 [file sensors-18-03960-s001.zip › sensors-377629-supplementary/annarbor/complex/154202627.png]

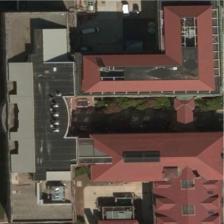

Supplement: Supplementary File 1 [file sensors-18-03960-s001.zip › sensors-377629-supplementary/annarbor/complex/154202689.jpg]

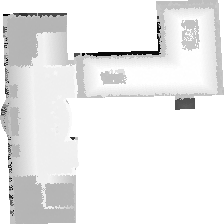

Supplement: Supplementary File 1 [file sensors-18-03960-s001.zip › sensors-377629-supplementary/annarbor/complex/154202689.png]

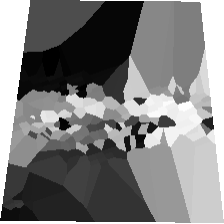

Supplement: Supplementary File 1 [file sensors-18-03960-s001.zip › sensors-377629-supplementary/annarbor/complex/382245085.png]

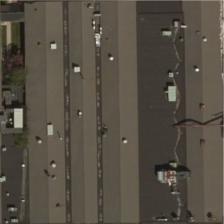

Supplement: Supplementary File 1 [file sensors-18-03960-s001.zip › sensors-377629-supplementary/annarbor/complex/384454221.jpg]

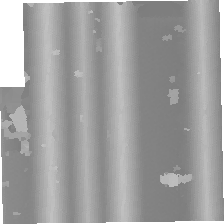

Supplement: Supplementary File 1 [file sensors-18-03960-s001.zip › sensors-377629-supplementary/annarbor/complex/384454221.png]

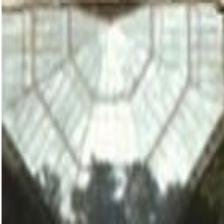

Supplement: Supplementary File 1 [file sensors-18-03960-s001.zip › sensors-377629-supplementary/annarbor/complex/412459961.jpg]

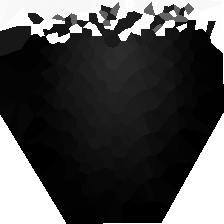

Supplement: Supplementary File 1 [file sensors-18-03960-s001.zip › sensors-377629-supplementary/annarbor/complex/412459961.png]

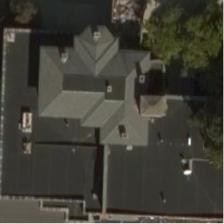

Supplement: Supplementary File 1 [file sensors-18-03960-s001.zip › sensors-377629-supplementary/annarbor/complex/453839878.jpg]

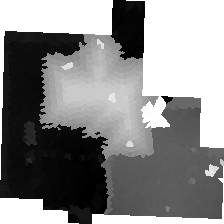

Supplement: Supplementary File 1 [file sensors-18-03960-s001.zip › sensors-377629-supplementary/annarbor/complex/453839878.png]

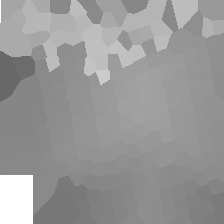

Supplement: Supplementary File 1 [file sensors-18-03960-s001.zip › sensors-377629-supplementary/annarbor/complex/454522854.png]

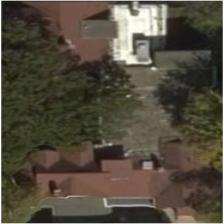

Supplement: Supplementary File 1 [file sensors-18-03960-s001.zip › sensors-377629-supplementary/annarbor/complex/471191924.jpg]

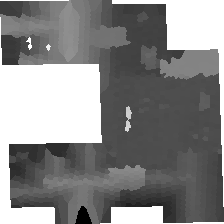

Supplement: Supplementary File 1 [file sensors-18-03960-s001.zip › sensors-377629-supplementary/annarbor/complex/471191924.png]

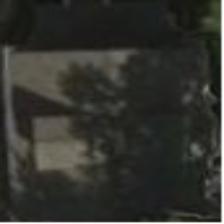

Supplement: Supplementary File 1 [file sensors-18-03960-s001.zip › sensors-377629-supplementary/annarbor/complex/473398703.jpg]

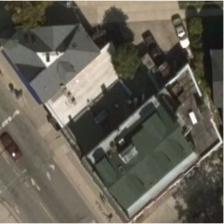

Supplement: Supplementary File 1 [file sensors-18-03960-s001.zip › sensors-377629-supplementary/annarbor/complex/480677946.jpg]

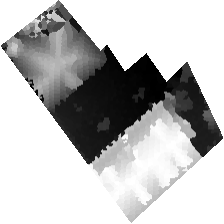

Supplement: Supplementary File 1 [file sensors-18-03960-s001.zip › sensors-377629-supplementary/annarbor/complex/480677946.png]

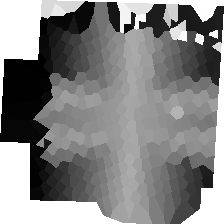

Supplement: Supplementary File 1 [file sensors-18-03960-s001.zip › sensors-377629-supplementary/annarbor/complex/482763995.png]

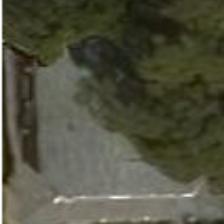

Supplement: Supplementary File 1 [file sensors-18-03960-s001.zip › sensors-377629-supplementary/annarbor/complex/484730958.jpg]

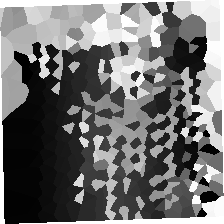

Supplement: Supplementary File 1 [file sensors-18-03960-s001.zip › sensors-377629-supplementary/annarbor/complex/485025731.png]

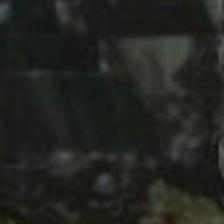

Supplement: Supplementary File 1 [file sensors-18-03960-s001.zip › sensors-377629-supplementary/annarbor/complex/485228253.jpg]

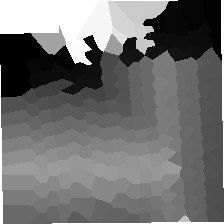

Supplement: Supplementary File 1 [file sensors-18-03960-s001.zip › sensors-377629-supplementary/annarbor/complex/485228253.png]

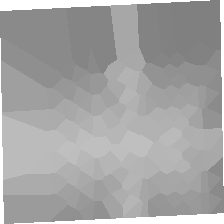

Supplement: Supplementary File 1 [file sensors-18-03960-s001.zip › sensors-377629-supplementary/annarbor/complex/485429900.png]

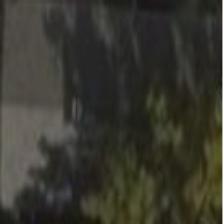

Supplement: Supplementary File 1 [file sensors-18-03960-s001.zip › sensors-377629-supplementary/annarbor/complex/488960360.jpg]

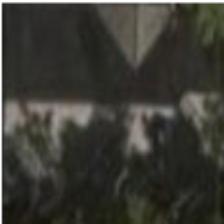

Supplement: Supplementary File 1 [file sensors-18-03960-s001.zip › sensors-377629-supplementary/annarbor/complex/490229136.jpg]

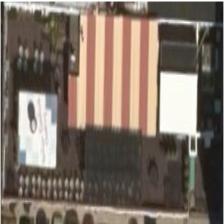

Supplement: Supplementary File 1 [file sensors-18-03960-s001.zip › sensors-377629-supplementary/annarbor/complex/493526512.jpg]

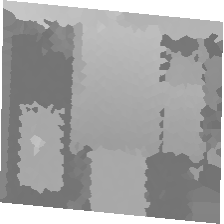

Supplement: Supplementary File 1 [file sensors-18-03960-s001.zip › sensors-377629-supplementary/annarbor/complex/493526512.png]

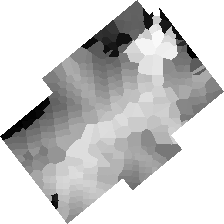

Supplement: Supplementary File 1 [file sensors-18-03960-s001.zip › sensors-377629-supplementary/annarbor/complex/493774999.png]

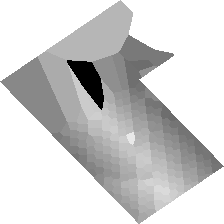

Supplement: Supplementary File 1 [file sensors-18-03960-s001.zip › sensors-377629-supplementary/annarbor/complex/493775000.png]

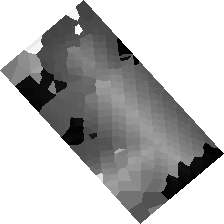

Supplement: Supplementary File 1 [file sensors-18-03960-s001.zip › sensors-377629-supplementary/annarbor/complex/493775001.png]

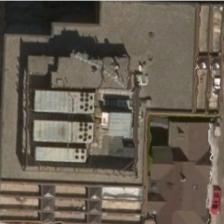

Supplement: Supplementary File 1 [file sensors-18-03960-s001.zip › sensors-377629-supplementary/annarbor/complex/499566752.jpg]

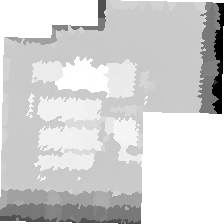

Supplement: Supplementary File 1 [file sensors-18-03960-s001.zip › sensors-377629-supplementary/annarbor/complex/499566752.png]

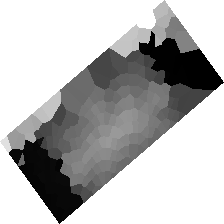

Supplement: Supplementary File 1 [file sensors-18-03960-s001.zip › sensors-377629-supplementary/annarbor/complex/502788286.png]

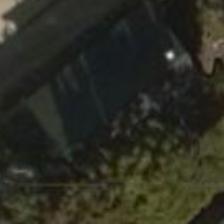

Supplement: Supplementary File 1 [file sensors-18-03960-s001.zip › sensors-377629-supplementary/annarbor/complex/502788297.jpg]

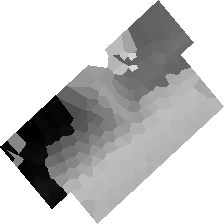

Supplement: Supplementary File 1 [file sensors-18-03960-s001.zip › sensors-377629-supplementary/annarbor/complex/502971279.png]

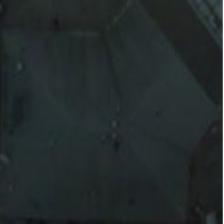

Supplement: Supplementary File 1 [file sensors-18-03960-s001.zip › sensors-377629-supplementary/annarbor/complex/503081138.jpg]

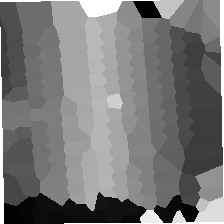

Supplement: Supplementary File 1 [file sensors-18-03960-s001.zip › sensors-377629-supplementary/annarbor/complex/504476154.png]

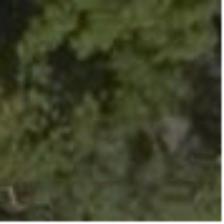

Supplement: Supplementary File 1 [file sensors-18-03960-s001.zip › sensors-377629-supplementary/annarbor/complex/519258369.jpg]

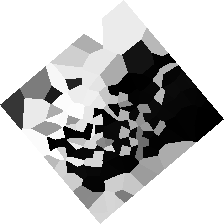

Supplement: Supplementary File 1 [file sensors-18-03960-s001.zip › sensors-377629-supplementary/annarbor/complex/519258369.png]

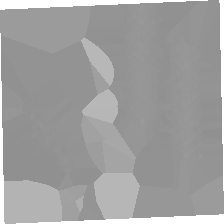

Supplement: Supplementary File 1 [file sensors-18-03960-s001.zip › sensors-377629-supplementary/annarbor/complex/562286287.png]

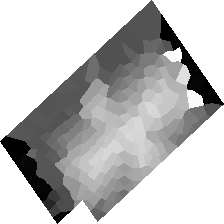

Supplement: Supplementary File 1 [file sensors-18-03960-s001.zip › sensors-377629-supplementary/annarbor/complex/591254004.png]

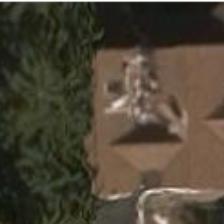

Supplement: Supplementary File 1 [file sensors-18-03960-s001.zip › sensors-377629-supplementary/annarbor/complex/612462707.jpg]

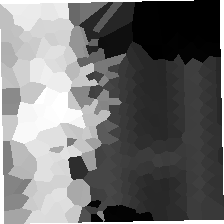

Supplement: Supplementary File 1 [file sensors-18-03960-s001.zip › sensors-377629-supplementary/annarbor/complex/612462707.png]

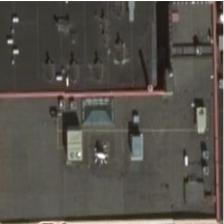

Supplement: Supplementary File 1 [file sensors-18-03960-s001.zip › sensors-377629-supplementary/annarbor/complex_flat/154202638.jpg]

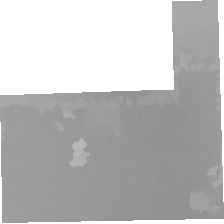

Supplement: Supplementary File 1 [file sensors-18-03960-s001.zip › sensors-377629-supplementary/annarbor/complex_flat/154202638.png]

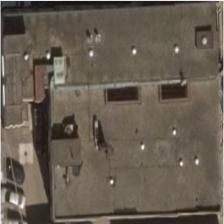

Supplement: Supplementary File 1 [file sensors-18-03960-s001.zip › sensors-377629-supplementary/annarbor/complex_flat/154202639.jpg]

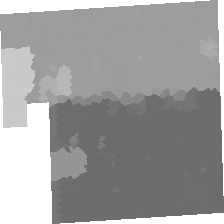

Supplement: Supplementary File 1 [file sensors-18-03960-s001.zip › sensors-377629-supplementary/annarbor/complex_flat/154202639.png]

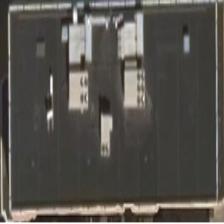

Supplement: Supplementary File 1 [file sensors-18-03960-s001.zip › sensors-377629-supplementary/annarbor/complex_flat/154202641.jpg]

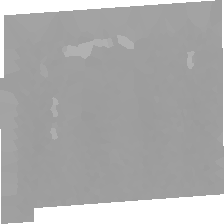

Supplement: Supplementary File 1 [file sensors-18-03960-s001.zip › sensors-377629-supplementary/annarbor/complex_flat/154202641.png]

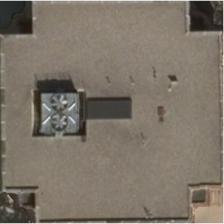

Supplement: Supplementary File 1 [file sensors-18-03960-s001.zip › sensors-377629-supplementary/annarbor/complex_flat/154203617.jpg]

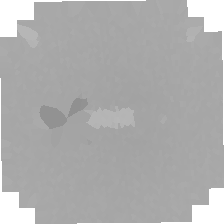

Supplement: Supplementary File 1 [file sensors-18-03960-s001.zip › sensors-377629-supplementary/annarbor/complex_flat/154203617.png]

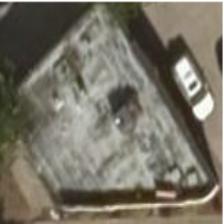

Supplement: Supplementary File 1 [file sensors-18-03960-s001.zip › sensors-377629-supplementary/annarbor/complex_flat/256667358.jpg]

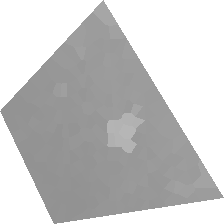

Supplement: Supplementary File 1 [file sensors-18-03960-s001.zip › sensors-377629-supplementary/annarbor/complex_flat/256667358.png]

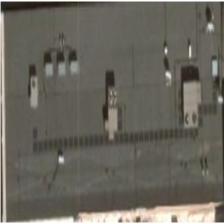

Supplement: Supplementary File 1 [file sensors-18-03960-s001.zip › sensors-377629-supplementary/annarbor/complex_flat/271052070.jpg]

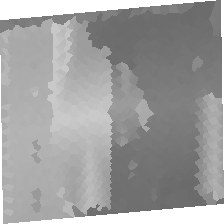

Supplement: Supplementary File 1 [file sensors-18-03960-s001.zip › sensors-377629-supplementary/annarbor/complex_flat/271052070.png]

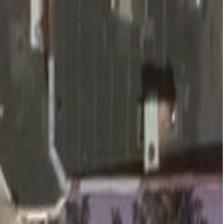

Supplement: Supplementary File 1 [file sensors-18-03960-s001.zip › sensors-377629-supplementary/annarbor/complex_flat/299946731.jpg]

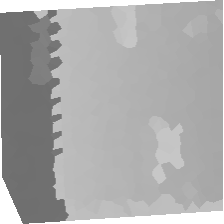

Supplement: Supplementary File 1 [file sensors-18-03960-s001.zip › sensors-377629-supplementary/annarbor/complex_flat/299946731.png]

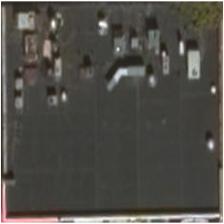

Supplement: Supplementary File 1 [file sensors-18-03960-s001.zip › sensors-377629-supplementary/annarbor/complex_flat/299946733.jpg]

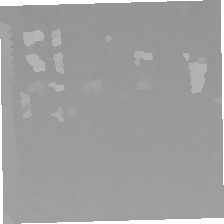

Supplement: Supplementary File 1 [file sensors-18-03960-s001.zip › sensors-377629-supplementary/annarbor/complex_flat/299946733.png]

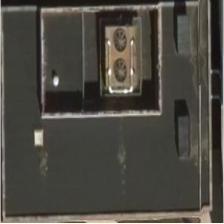

Supplement: Supplementary File 1 [file sensors-18-03960-s001.zip › sensors-377629-supplementary/annarbor/complex_flat/300676081.jpg]

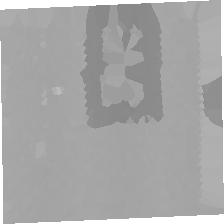

Supplement: Supplementary File 1 [file sensors-18-03960-s001.zip › sensors-377629-supplementary/annarbor/complex_flat/300676081.png]

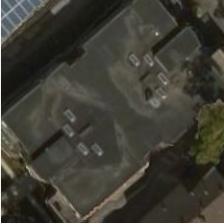

Supplement: Supplementary File 1 [file sensors-18-03960-s001.zip › sensors-377629-supplementary/annarbor/complex_flat/302138919.jpg]

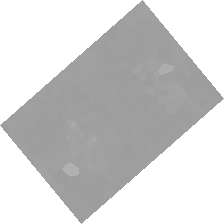

Supplement: Supplementary File 1 [file sensors-18-03960-s001.zip › sensors-377629-supplementary/annarbor/complex_flat/302138919.png]

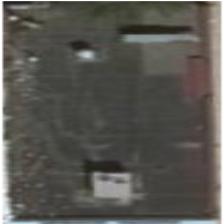

Supplement: Supplementary File 1 [file sensors-18-03960-s001.zip › sensors-377629-supplementary/annarbor/complex_flat/302181016.jpg]

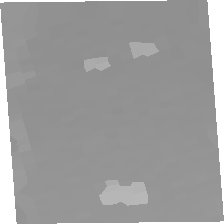

Supplement: Supplementary File 1 [file sensors-18-03960-s001.zip › sensors-377629-supplementary/annarbor/complex_flat/302181016.png]

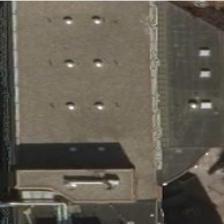

Supplement: Supplementary File 1 [file sensors-18-03960-s001.zip › sensors-377629-supplementary/annarbor/complex_flat/303632984.jpg]

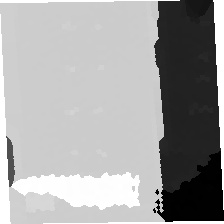

Supplement: Supplementary File 1 [file sensors-18-03960-s001.zip › sensors-377629-supplementary/annarbor/complex_flat/303632984.png]

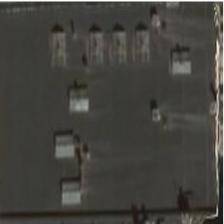

Supplement: Supplementary File 1 [file sensors-18-03960-s001.zip › sensors-377629-supplementary/annarbor/complex_flat/366476391.jpg]

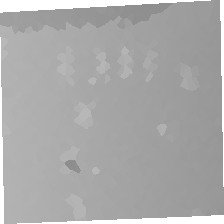

Supplement: Supplementary File 1 [file sensors-18-03960-s001.zip › sensors-377629-supplementary/annarbor/complex_flat/366476391.png]

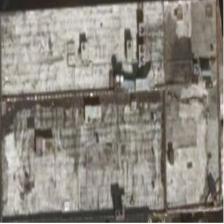

Supplement: Supplementary File 1 [file sensors-18-03960-s001.zip › sensors-377629-supplementary/annarbor/complex_flat/366476392.jpg]

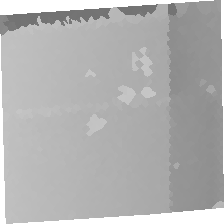

Supplement: Supplementary File 1 [file sensors-18-03960-s001.zip › sensors-377629-supplementary/annarbor/complex_flat/366476392.png]

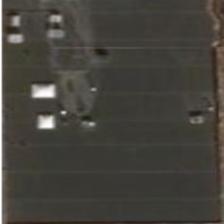

Supplement: Supplementary File 1 [file sensors-18-03960-s001.zip › sensors-377629-supplementary/annarbor/complex_flat/377804674.jpg]

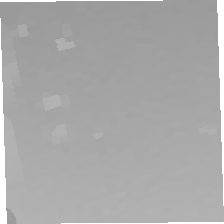

Supplement: Supplementary File 1 [file sensors-18-03960-s001.zip › sensors-377629-supplementary/annarbor/complex_flat/377804674.png]

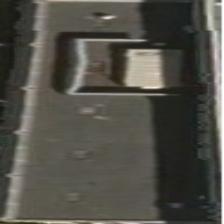

Supplement: Supplementary File 1 [file sensors-18-03960-s001.zip › sensors-377629-supplementary/annarbor/complex_flat/382245085.jpg]

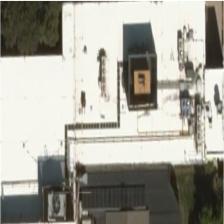

Supplement: Supplementary File 1 [file sensors-18-03960-s001.zip › sensors-377629-supplementary/annarbor/complex_flat/384454216.jpg]

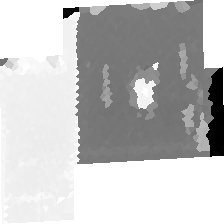

Supplement: Supplementary File 1 [file sensors-18-03960-s001.zip › sensors-377629-supplementary/annarbor/complex_flat/384454216.png]

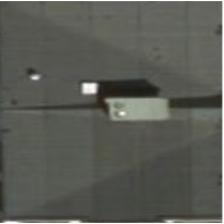

Supplement: Supplementary File 1 [file sensors-18-03960-s001.zip › sensors-377629-supplementary/annarbor/complex_flat/384454218.jpg]

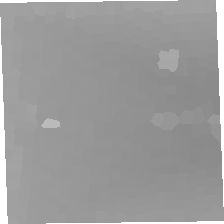

Supplement: Supplementary File 1 [file sensors-18-03960-s001.zip › sensors-377629-supplementary/annarbor/complex_flat/384454218.png]

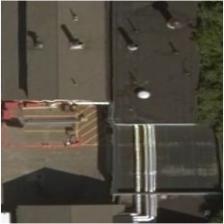

Supplement: Supplementary File 1 [file sensors-18-03960-s001.zip › sensors-377629-supplementary/annarbor/complex_flat/384455072.jpg]

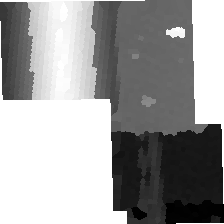

Supplement: Supplementary File 1 [file sensors-18-03960-s001.zip › sensors-377629-supplementary/annarbor/complex_flat/384455072.png]

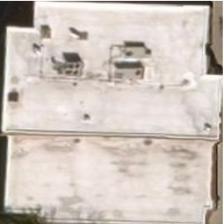

Supplement: Supplementary File 1 [file sensors-18-03960-s001.zip › sensors-377629-supplementary/annarbor/complex_flat/419321069.jpg]

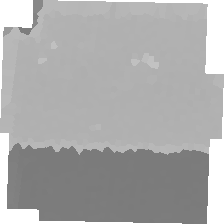

Supplement: Supplementary File 1 [file sensors-18-03960-s001.zip › sensors-377629-supplementary/annarbor/complex_flat/419321069.png]

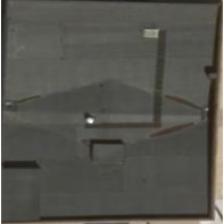

Supplement: Supplementary File 1 [file sensors-18-03960-s001.zip › sensors-377629-supplementary/annarbor/complex_flat/443277789.jpg]

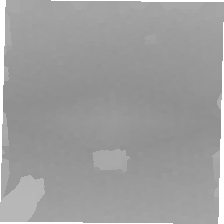

Supplement: Supplementary File 1 [file sensors-18-03960-s001.zip › sensors-377629-supplementary/annarbor/complex_flat/443277789.png]

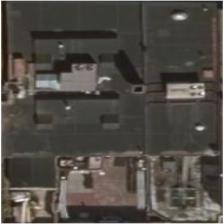

Supplement: Supplementary File 1 [file sensors-18-03960-s001.zip › sensors-377629-supplementary/annarbor/complex_flat/453538407.jpg]

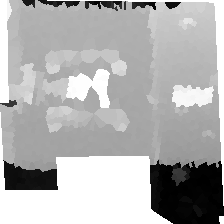

Supplement: Supplementary File 1 [file sensors-18-03960-s001.zip › sensors-377629-supplementary/annarbor/complex_flat/453538407.png]

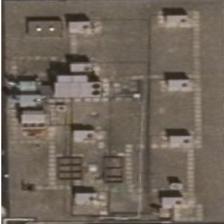

Supplement: Supplementary File 1 [file sensors-18-03960-s001.zip › sensors-377629-supplementary/annarbor/complex_flat/453538408.jpg]

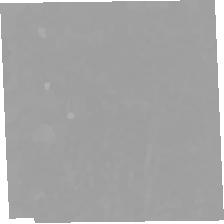

Supplement: Supplementary File 1 [file sensors-18-03960-s001.zip › sensors-377629-supplementary/annarbor/complex_flat/453538408.png]

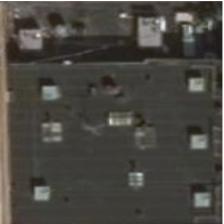

Supplement: Supplementary File 1 [file sensors-18-03960-s001.zip › sensors-377629-supplementary/annarbor/complex_flat/453674971.jpg]

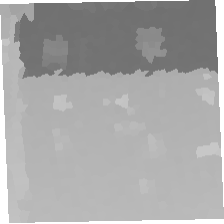

Supplement: Supplementary File 1 [file sensors-18-03960-s001.zip › sensors-377629-supplementary/annarbor/complex_flat/453674971.png]

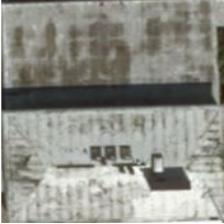

Supplement: Supplementary File 1 [file sensors-18-03960-s001.zip › sensors-377629-supplementary/annarbor/complex_flat/457363252.jpg]

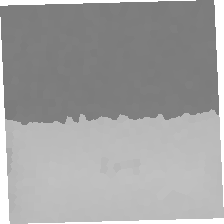

Supplement: Supplementary File 1 [file sensors-18-03960-s001.zip › sensors-377629-supplementary/annarbor/complex_flat/457363252.png]

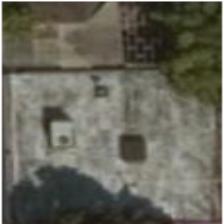

Supplement: Supplementary File 1 [file sensors-18-03960-s001.zip › sensors-377629-supplementary/annarbor/complex_flat/457363259.jpg]

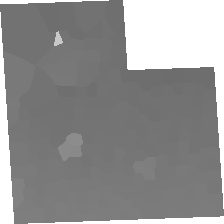

Supplement: Supplementary File 1 [file sensors-18-03960-s001.zip › sensors-377629-supplementary/annarbor/complex_flat/457363259.png]

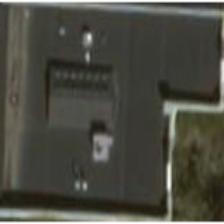

Supplement: Supplementary File 1 [file sensors-18-03960-s001.zip › sensors-377629-supplementary/annarbor/complex_flat/458822675.jpg]

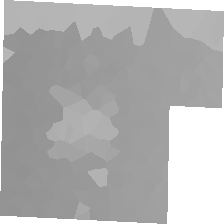

Supplement: Supplementary File 1 [file sensors-18-03960-s001.zip › sensors-377629-supplementary/annarbor/complex_flat/458822675.png]
